# Diverse Molecular Mechanisms Contribute to Differential Expression of Human Duplicated Genes

**DOI:** 10.1093/molbev/msab131

**Published:** 2021-05-01

**Authors:** Colin J Shew, Paulina Carmona-Mora, Daniela C Soto, Mira Mastoras, Elizabeth Roberts, Joseph Rosas, Dhriti Jagannathan, Gulhan Kaya, Henriette O’Geen, Megan Y Dennis

**Affiliations:** 1 Genome Center, University of California Davis, CA, USA; 2 Integrative Genetics and Genomics Graduate Group, University of California Davis, CA, USA; 3 MIND Institute, University of California, Davis, CA, USA; 4 Autism Research Training Program, University of California, Davis, CA, USA; 5 Postbaccalaureate Research Education Program, University of California, Davis, CA, USA; 6 Department of Biochemistry & Molecular Medicine, University of California, Davis, CA, USA

**Keywords:** gene duplication, gene regulation, primate evolution

## Abstract

Emerging evidence links genes within human-specific segmental duplications (HSDs) to traits and diseases unique to our species. Strikingly, despite being nearly identical by sequence (>98.5%), paralogous HSD genes are differentially expressed across human cell and tissue types, though the underlying mechanisms have not been examined. We compared cross-tissue mRNA levels of 75 HSD genes from 30 families between humans and chimpanzees and found expression patterns consistent with relaxed selection on or neofunctionalization of derived paralogs. In general, ancestral paralogs exhibited greatest expression conservation with chimpanzee orthologs, though exceptions suggest certain derived paralogs may retain or supplant ancestral functions. Concordantly, analysis of long-read isoform sequencing data sets from diverse human tissues and cell lines found that about half of derived paralogs exhibited globally lower expression. To understand mechanisms underlying these differences, we leveraged data from human lymphoblastoid cell lines (LCLs) and found no relationship between paralogous expression divergence and post-transcriptional regulation, sequence divergence, or copy-number variation. Considering *cis*-regulation, we reanalyzed ENCODE data and recovered hundreds of previously unidentified candidate CREs in HSDs. We also generated large-insert ChIP-sequencing data for active chromatin features in an LCL to better distinguish paralogous regions. Some duplicated CREs were sufficient to drive differential reporter activity, suggesting they may contribute to divergent *cis*-regulation of paralogous genes. This work provides evidence that *cis*-regulatory divergence contributes to novel expression patterns of recent gene duplicates in humans.

## Introduction

Gene duplication occurs universally and is considered a major source of evolutionary novelty; across eukaryotes, over 30% of genes are thought to have arisen from duplications (Zhang 2003). Although many duplicated genes rapidly become pseudogenes, some may share and maintain important ancestral functions via subfunctionalization, or gain novel functions entirely (neofunctionalization) (Lynch 2000). Expression divergence is likely integral to the survival of paralogous genes, as spatiotemporal partitioning of function places both daughter paralogs under purifying selection helping them escape pseudogenization (Force 1999; Rodin and Riggs 2003; Rodin et al. 2005). This may be the primary driver of duplicate gene retention, as gene regulation can be altered relatively easily while coding sequences remain intact (Ohno 1970). For example, mouse *Hoxa1* and *Hoxb1* genes are functionally redundant but partitioned by expression, with normal development possible from a single gene under the control of regulatory elements from both paralogs (Tvrdik and Capecchi 2006). On a genome-wide scale, substantial expression divergence has been observed in vertebrates following whole-genome duplications specific to teleost and salmonid fishes (Kassahn et al. 2009; Braasch et al. 2016; Lien et al. 2016; Varadharajan et al. 2018). Meta-analysis suggests that across all of these species, selection on gene-expression levels appears relaxed in one of the paralogs (Sandve et al. 2018). However, segmental duplications (SDs, regions defined as having >90% sequence similarity and being at least 1 kilobase pair [kb] in size (Bailey 2002)) occur more commonly in vertebrates than whole-genome duplications and concomitantly generate structural rearrangements, potentially facilitating regulatory divergence and duplicate retention (Rodin et al. 2005). Although comparative studies characterizing expression divergence of duplicated genes in humans, mice, and yeast have identified broad patterns of dosage sharing among daughter paralogs (Qian et al. 2010; Lan and Pritchard 2016), younger, human-specific duplications have yet to be analyzed in this light. Further, no molecular explanations have been provided for the observed expression changes between paralogs.

Great apes have experienced a surge of SDs in the last ∼10 My that arose primarily interspersed throughout the genome and potentially contribute to phenotypic differences observed between these closely related species (Prado-Martinez et al. 2013). Human-specific SDs (HSDs), which arose in the last ∼6 My following the split of the human and chimpanzee lineages, contain genes that have compelling associations with neurodevelopmental features (Charrier et al. 2012; Dennis et al. 2012; Florio et al. 2015; Fiddes et al. 2018; Suzuki et al. 2018; Heide et al. 2020) and disorders (Dennis and Eichler 2016; Dennis et al. 2017; Ishiura et al. 2019). Historically, such young duplications have been poorly resolved in genome assemblies due to their high sequence similarity. Recent sequencing efforts targeted to HSDs have generated high-quality assemblies for many of these loci (Steinberg et al. 2012; Antonacci et al. 2014; O’Bleness et al. 2014; Dennis et al. 2017) resulting in the discovery of at least 30 gene families containing >80 paralogs uniquely duplicated and existing in >90% of humans. Most derived HSD genes encode putatively functional proteins and exhibit divergent expression patterns relative to ancestral paralogs across numerous primary tissues, despite HSDs being nearly identical by sequence (on average ∼99.5%) (Dennis et al. 2017). Although there are examples of HSD genes exapting novel promoters and exons at the site of insertion (Dougherty et al. 2017), this cannot explain expression divergence that exists among whole-gene duplications. Differential regulation may be intertwined with associations of species-specific active chromatin modifications at SD loci (Giannuzzi et al. 2014), but historical reference errors and computational challenges in short-read mapping to highly similar sequences has resulted in poorly annotated epigenetic information at duplicated loci (Chung et al. 2011; Ebbert et al. 2019).

In this study, we characterized patterns of regulatory divergence observed for HSD genes between humans and chimpanzees by quantifying cross-tissue conservation of orthologous gene expression. We found that even the youngest of duplicate genes have diverged in expression and, by comparing expression divergence between ancestral and derived paralogs, have begun to infer changes to HSD gene function. We leveraged genomic and epigenomic data from hundreds of human lymphoblastoid cell lines (LCLs) to identify differentially expressed (DE) ancestral-derived gene pairs and examined potential molecular contributors to paralogous expression divergence, including copy-number (CN) variation, post-transcriptional regulation, and *cis*-regulatory changes. Finally, we surveyed the active chromatin “landscape” of HSDs by reanalyzing ENCODE histone modification chromatin immunoprecipitation sequence (ChIP-seq) data, produced a novel “longer-read” ChIP-seq data set to improve the unique alignment rate in SDs, and functionally validated candidate *cis*-regulatory elements (cCREs) via a reporter assay. Overall, our work demonstrates that *cis*-regulatory divergence, among other mechanisms, drives differential expression following gene duplication and that useful regulatory information can be rescued from existing data sets for duplicated loci.

## Results

### Conservation of HSD Gene Expression following Duplication

**Fig. 1.**
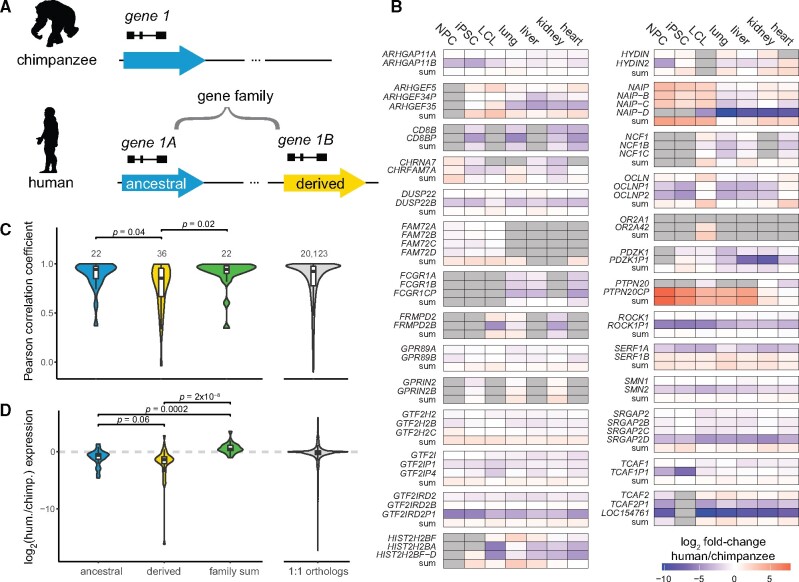
Expression patterns of HSD genes between species. (*A*) Illustration of genes residing within HSDs; the ancestral paralog (blue) corresponds to the chimpanzee ortholog, while derived paralogs (yellow) are human-specific. The ancestral and derived genes comprise a gene family. (*B*) HSD gene expression differences between humans and chimpanzees in three cell lines and four primary tissues. Cells are colored by the log**_2_-**fold change of human versus chimpanzee expression. Gray cells indicate nonexpressed genes. Note, *PTPN20CP* is expressed many fold higher than *PTPN20* and the chimpanzee ortholog, but both paralogs are lowly expressed (<2 TPM) in most samples assayed. Differential expression results are provided in supplementary table S5, Supplementary Material online. (*C, D*) Comparison of human gene expression with chimpanzee orthologs. Violin and box plots represent cross-tissue expression correlations (*C*) and relative expression levels (log_2_ ratio of human (hum.) versus chimpanzee (chimp.) expression, averaged across all cell and tissue types; supplementary fig. S1, Supplementary Material online) (*D*). HSD genes of known evolutionary status were classified as ancestral (blue) or derived (yellow) and compared with the aggregated gene family expression (green). *P*-values were calculated from Dunn’s test following a Kruskal–Wallis test. Expression correlations of one-to-one orthologs are visualized for reference.

To assess the evolutionary trajectory of recent human duplicated genes, we quantified expression of 75 HSD genes from 30 gene families for which high-confidence sequences were available (Dennis et al. 2017) (supplementary table S1, Supplementary Material online). The SDs comprising these genes duplicated in an interspersed manner, typically hundreds of kilobases away from the ancestral locus (median 871 kb; mean 6 Mb (Dennis et al. 2017)), with two of the 30 gene families residing on separate chromosomes. Each HSD gene family corresponded to a single-copy chimpanzee ortholog and multiple (2–4) human paralogs. If known, we classified the human paralog syntenic with the chimpanzee gene as ancestral and the human-specific paralog(s) as derived (fig. 1*A*, supplementary table S1, Supplementary Material online). To interpret the evolutionary fate of these genes, we compared expression of HSD paralogs (individual or summed) to chimpanzee orthologs using mRNA-sequencing (RNA-seq) data from three cell lines and four primary tissues (Khan et al. 2013; Pavlovic et al. 2018; Marchetto et al. 2019; Blake et al. 2020) using a lightweight mapping approach that shows high accuracy for paralogous genes (Soneson et al. 2015; Patro et al. 2017). Derived HSD paralogs tended to exhibit lower expression than the chimpanzee ortholog, summed family expression was mostly higher, and ancestral paralogs were less likely to be DE (9/21 expressed ancestral genes showed no differential expression across all cell/tissue types vs. 6/37 of expressed derived genes; *p *=* *0.028, Fisher’s Exact Test) (fig. 1*B*, supplementary table S2, Supplementary Material online). Altogether, these results suggest that ancestral genes tend to retain their expression patterns, while derived paralogs diverge and typically lose expression.

We next considered expression correlation across the four tissue types and three cell lines as a proxy for expression conservation between human genes and their chimpanzee orthologs. Our expectation was that in the case of subfunctionalization, the summed expression of all HSD paralogs would correlate best with chimpanzee expression, while all individual paralogs would be less correlated; and in the cases of pseudogenization or neofunctionalization, a single paralog would exhibit high correlation with chimpanzee expression (Braasch et al. 2018; Sandve et al. 2018). We found that derived HSD paralogs exhibited significantly lower expression conservation than ancestral paralogs or summed expression, which were statistically equivalent (Kruskal–Wallis test followed by Dunn’s test, Benjamini–Hochberg adjusted *P *<* *0.05; fig. 1*C*, supplementary table S3, Supplementary Material online). This pattern is broadly consistent with maintenance of the ancestral paralog and divergence of expression patterns of the others via relaxed selection or neofunctionalization. Further, the most conserved gene in each family was usually the ancestral paralog (14/22 of known status, *P *<* *0.001, hypergeometric test). Nevertheless, eight derived paralogs showed strongest conservation of expression with chimpanzee orthologs and represent candidates for supplanting functions of their ancestral gene. For example, *SERF1B* exhibited higher expression correlation with chimpanzee than the ancestral *SERF1A* (Pearson’s *r* of 0.81 and 0.74, respectively), while *SERF1A* expression was reduced relative to chimpanzee in lung, LCLs, and induced pluripotent stem cells (iPSCs) (supplementary tables S2 and S3, Supplementary Material online). A few gene families (such as *CD8B*, *GTF2IRD2*, and *NAIP*) displayed expression patterns consistent with subfunctionalization, as their summed expression correlated better with that of chimpanzee than any individual paralog; however, in these cases the difference was small (difference in Pearson’s *r *<* *0.05 between sum and most correlated paralog). We next considered relative expression levels between species and found that across tissues, ancestral paralogs trended toward higher expression than derived paralogs (Kruskal–Wallis test followed by Dunn’s test, Benjamini–Hochberg adjusted *P *=* *0.058; fig. 1*D* and supplementary fig. S1*A*, Supplementary Material online). As expected, summed HSD paralog expression was significantly higher than ancestral or derived paralogs alone. Finally, we calculated the tissue specificity index τ (Yanai et al. 2005) for HSD genes and one-to-one orthologs and found no significant differences between ancestral and derived genes (supplementary fig. S1*B*, supplementary table S3, Supplementary Material online). Taken together, our analyses provide little evidence for subfunctionalization of HSD genes and are consistent with derived paralogs experiencing relaxed selection.

These results are concordant with our previous finding that derived paralogs globally show a reduction of expression relative to ancestral paralogs, with some exceptions, across diverse human tissues and cell lines from the Genotype-Tissue Expression project (Dennis et al. 2017). To validate this with a strict alignment-based approach, we used long-read PacBio isoform sequencing (Iso-Seq) data, which maps to paralogous loci with higher confidence, from a panel of 24 human biosamples and cell lines (Encyclopedia of DNA Elements [ENCODE] project). From this, we again found globally reduced expression of derived paralogs: 21/41 derived genes were expressed at a level below their ancestral paralog, while two derived genes were higher (*P* < 0.05, Wilcoxon Signed-Rank test with Benjamini–Hochberg correction; supplementary fig. S2, Supplementary Material online). Though results should be interpreted cautiously given the low read depth and small number of replicates for each biosample, we also observed some derived paralogs exhibit greater expression than the ancestral paralog in individual tissues or cell types; one compelling example was diverged expression of *ARHGAP11B* in excitatory neurons, which matches published findings related to the novel function of this gene in the human cortex (Florio et al. 2015; Kalebic et al. 2018; Heide et al. 2020).

### Expression of HSD Paralogs in LCLs

**Fig. 2.**
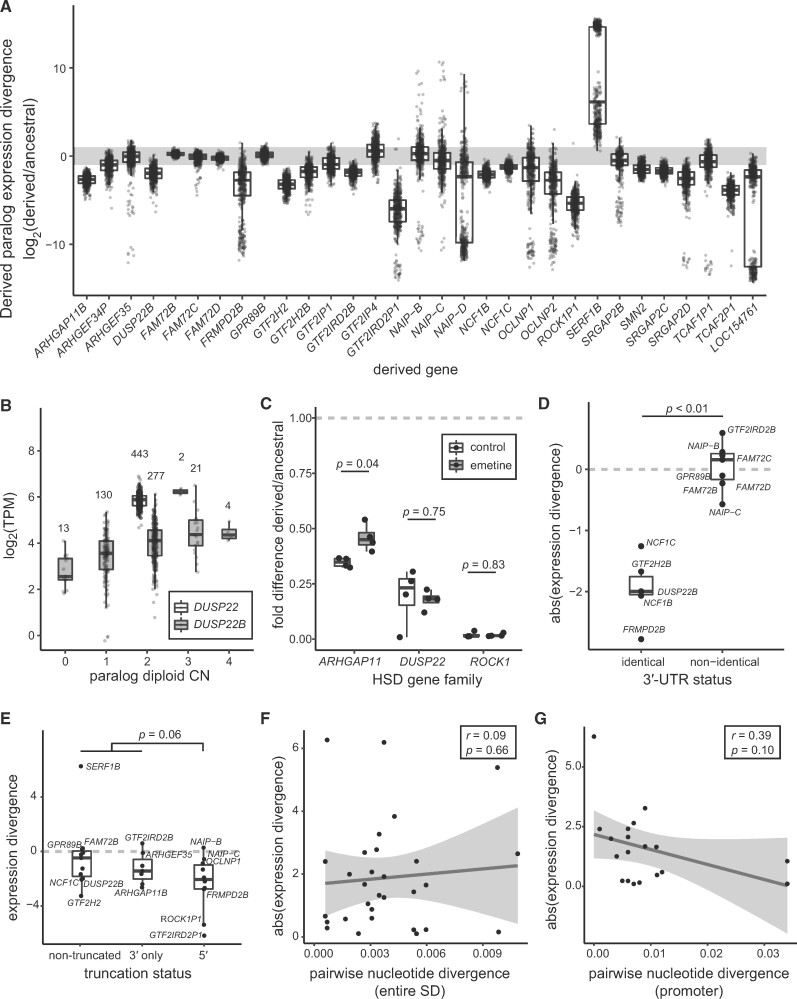
Differential expression of HSD genes in human LCLs. (*A*) Expression divergence of derived genes is plotted as the log_2_ ratio of median derived and ancestral expression for families with at least one LCL-expressed paralog. Each point represents an LCL from the Geuvadis consortium (total *N* = 445) (Lappalainen et al. 2013). The gray bar indicates a 2-fold expression difference. (*B*) Expression values of ancestral *DUSP22* (white) and derived *DUSP22B* (gray), stratified by CN. The number of individuals represented in each CN category is denoted over each boxplot. (*C*) Derived/ancestral fold-differences in expression determined from paralog-specific qPCR in control (white) and NMD-inhibited (gray) LCLs (*N* = 4). Statistical significance in panels *C–E* was assessed with a Wilcoxon signed-rank test. (*D*) Absolute value of expression divergence of ancestral-derived gene pairs, stratified by identical or nonidentical 3′ UTRs. (*E*) Comparison of expression divergence across truncation status for all expressed ancestral-derived gene pairs. (*F, G*) Scatterplot of the absolute value of expression divergence versus pairwise nucleotide identity for all expressed ancestral-derived gene pairs for whole duplicons (*F*) and promoters (*G*). Regression lines (black) and 95% confidence intervals are shown, along with the Pearson correlation coefficient (*r*) and significance of the regression slope (*p*).

We next focused on LCLs to gain a more detailed understanding of HSD expression patterns across hundreds of individuals with matched genomic data. We estimated transcript abundance using RNA-seq data from 462 human LCLs (Lappalainen et al. 2013) (supplementary table S4, Supplementary Material online) and found high concordance with expression estimates from Iso-Seq data from the LCL GM12878 (Pearson’s *r *=* *0.94 for 72 genes common to both analyses). We determined that over half (43/75) of HSD paralogs were expressed above one transcript per million (TPM), with the most highly expressed genes including *ARHGAP11A; ROCK1;* the adjacent *GTF2I* and *NCF1* families; and the *DUSP22* family, whose derived paralog *DUSP22B* is missing from the human reference (GRCh38) (Dennis et al. 2017). Comparing expression profiles within gene families, derived and ancestral paralogs globally showed divergent expression levels. In families with at least one expressed gene, all 31 derived genes showed significant differences from their ancestral counterpart, with a median TPM difference greater than 2-fold in 20 of these. As was found across other cells/tissues, in most cases (25/31) the derived gene had lower expression, which we confirmed for three highly expressed gene families with RT-qPCR and Iso-Seq data (fig. 2*A*, supplementary fig. S3, Supplementary Material online, and supplementary table S5, Supplementary Material online). We noted that some paralogs exhibited high- or low-clustered values for derived to ancestral expression ratios, caused by lack of expression of one of the genes in a subset of individuals. This could not be reconciled as CN or population of origin differences (supplementary fig. S4, Supplementary Material online), sex, or technical effects due to sequencing depth or sequencing facility (data not shown). Altogether, these results indicate that paralogous HSD genes show divergent expression patterns in LCLs across hundreds of diverse samples.

### CN Variation and HSD Expression

While the genes in this study were chosen for being nearly fixed in modern human populations (Dennis et al. 2017), SD loci are subject to recurrent rearrangement and consequently exhibit varying degrees of CN polymorphism. Understanding that CN variation can alter gene expression levels (Stranger et al. 2007), we sought to characterize its impact on differential expression of HSD genes. After performing paralog-specific CN genotyping (Shen and Kidd 2020) of a subset of individuals for which 1000 Genomes Illumina sequences were available (*N* = 445), we found gene expression was positively associated with CN in about half (28/55) of genes in expressed families (supplementary table S6, Supplementary Material online), indicating that higher CN often but not always results in increased expression. Notably, derived genes tended to have higher CN (averaging 1.2-fold higher than ancestral over all genes), but lower expression overall. We next used linear regression to remove the effect of CN from these comparisons and found 23/25 derived paralogs were still DE with respect to the ancestral (six were not tested due to paralog-specific effects of CN; supplementary table S5, Supplementary Material online). For example, while expression of *DUSP22B* was significantly associated with CN, these effects were insufficient to explain DE relative to *DUSP22* (fig. 2*B*). Thus, while CN differences alter the mRNA abundance of HSD paralogs, they do not provide an explanation for overall DE of these genes.

### Post-transcriptional Regulation of HSD Genes

In order to determine if paralogous expression differences are driven by post-transcriptional regulation, we next considered whether HSD transcripts were being processed as nonfunctional pseudogenes. In this scenario, paralogs might be equally transcribed but differentially subject to degradation via nonsense-mediated decay (NMD). To test this, we compared gene expression using available RNA-seq data from human NMD-deficient LCLs (*N* = 4) against controls (*N* = 2) (Nguyen et al. 2012) and found no HSD genes among identified DE genes. We also assessed directly if the ratio of derived to ancestral expression changed for each HSD gene family between NMD-deficient LCLs and controls and found no significant differences, though sample sizes were likely limiting (supplementary fig. S5, Supplementary Material online). This result was largely recapitulated by paralog-specific RT-qPCR for three DE HSD genes families (*ARHGAP11, DUSP22*, and *ROCK1*) in four LCLs treated with the NMD-inhibiting drug emetine. Ratios of *ROCK1P1/ROCK1* and *DUSP22B/DUSP22* expression were unaltered by emetine treatment, while *ARHGAP11B*/*ARHGAP11A* expression ratio increased closer to one, consistent with NMD affecting *ARHGAP11B*, though not completely “rescuing” derived expression levels to equal that of the ancestral (fig. 2*C*). *ARHGAP11B* is a 3′ truncation of *ARHGAP11A*, potentially explaining differences in transcript stability. Altogether, these results suggest that while NMD may alter steady-state expression levels of some HSD genes, it is not a primary driver of their differential expression.

We also examined HSD 3′ untranslated regions (UTRs) for recognition sites of miRNAs expressed in LCLs (Lappalainen et al. 2013) (*N* = 13 3′ UTRs of expressed gene families; mean 94 binding sites per UTR) using TargetScan (Agarwal et al. 2015). Although miRNA binding sites were nearly identical between paralogs, we unexpectedly observed significantly greater expression divergence between paralogs with identical 3′ UTRs (*N* = 5) from those that differed (*N* = 7) (Wilcoxon signed-rank test *P* < 0.01, fig. 2*D*). While these data cannot rule out a role for miRNAs in HSD transcriptional regulation, this mechanism does not explain observed differential expression of expressed gene families with identical 3′ UTRs, such as *DUSP22* and *NCF1.*

### Role of *Cis*-Regulation in HSD Differential Expression

We next aimed to determine if *cis*-regulatory changes contribute to expression divergence of HSDs. Because SDs often generate gene truncations and fusions with adjacent transcribed sequences (Dougherty et al. 2017), we reasoned that gains or losses of promoters or UTRs would likely cause large changes in gene expression. We compared relative expression by truncation status (5′-, 3′-, or nontruncated) of all derived genes in expressed families to their ancestral paralogs. Ancestral and derived genes had more similar expression levels in nontruncating duplications, while truncated genes tended to be less expressed than their ancestral paralogs, particularly 5′ truncations compared to all other HSD genes (*P *=* *0.057, *t*-test; fig. 2*E*), in concordance with previous findings (Dougherty et al. 2018). While we may have limited power to detect differences with our small number of genes, these results hint that promoter activity is an important determinant of differential expression patterns. Considering sequence-level changes more broadly, however, we observed no relationship between expression divergence and pairwise nucleotide divergence across entire duplicons or within promoters (fig. 2*F* and *G*).

**Fig. 3.**
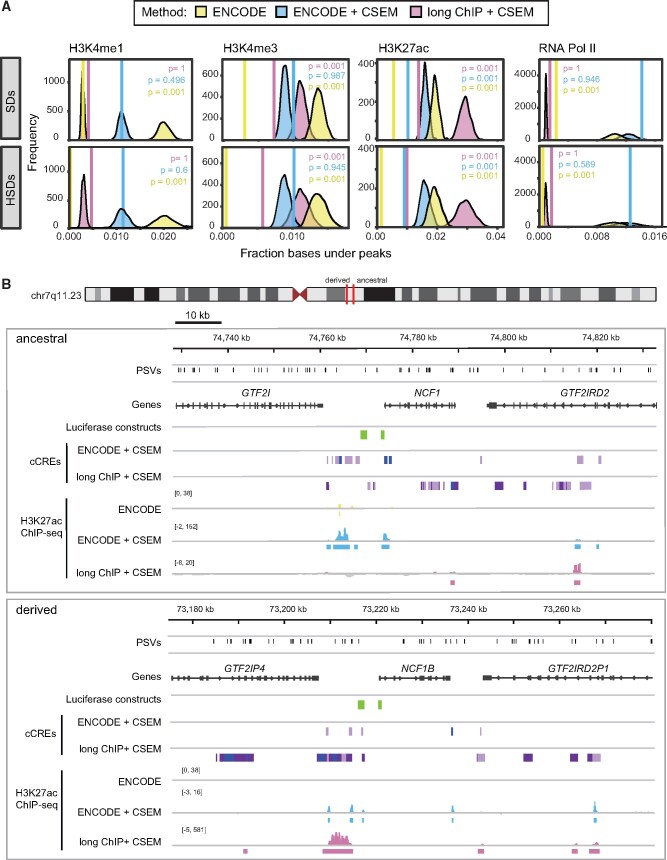
Depletion and recovery of ChIP peaks in SDs. (*A*) The fraction of bases covered by peaks (solid vertical line) was computed in SDs (top) and HSDs (SDs >98% sequence identity, bottom) for three ChIP-seq peak discovery approaches: publicly available ENCODE peaks (yellow), peaks from multimapping and CSEM allocation of ENCODE raw data (blue), and peaks from multimapping and CSEM allocation of large-insert ChIP-sequencing (“long ChIP”) data from this publication (magenta). SD coordinates were permuted 1000 times within the human reference (GRCh38), and an expected distribution of the fraction of bases covered was generated. Empirical one-sided *P*-values for depletion are indicated in each graph. (*B*) Chromatin landscape at the chromosome 7q11.23 HSD locus. The ancestral locus (top) and one of its derived loci (bottom) are shown with PSVs (black), genes (gray), and luciferase-tested regions (green). cCREs were identified with an 8-state ChromHMM model of GM12878 H3K4me3, H3K4me1, and H3K27ac data from multimapping reanalysis of ENCODE and long ChIP data after CSEM allocation (enhancer states in light and dark purple and promoter states in blue). H3K27ac ChIP-seq data (signal and peak calls) are also shown in yellow, blue, and magenta for published ENCODE, reanalyzed ENCODE + CSEM, and long ChIP + CSEM, respectively.

Given that the vast majority of paralog-specific variants (PSVs) distinguishing HSDs are unlikely to be functional, we used publicly available chromatin immunoprecipitation sequencing (ChIP-seq) data sets from the ENCODE project (The ENCODE Project Consortium 2012; Davis et al. 2018) to identify likely CREs (H3K4me3, H3K4me1, H3K27ac, and RNA PolII) in a single LCL for which a wealth of functional genomic data exists (GM12878). In each data set, we observed a lower density of bases covered by peaks in SDs (>90% similarity) and HSDs (>98% similarity) compared to randomly sampled regions of equivalent size (empirical *P* = 0.001, *N *= 1000 replicates; fig. 3*A*, in yellow). We posit, as others have previously (Chung et al. 2011; McVicker et al. 2013; Giannuzzi et al. 2014), that this discrepancy is an artifact of the high sequence similarity of SDs, with reads originating from these regions often discarded when mapping to multiple locations of the genome.

To recover this missing information, we implemented a pipeline that allowed reads to align to multiple locations in the genome and then, using CSEM (Chung et al. 2011), iteratively weighted alignments based on the nearby unique mapping rate. Selecting the most likely alignment to allocate a read (i.e., mapping position with the highest posterior probability), we improved peak discovery in SDs and HSDs for the aforementioned chromatin features, erasing the depletion for all but H3K27ac, which was still substantially improved (fig. 3*A*, in blue). The peaks we discovered largely overlapped with the ENCODE peaks, though RNA PolII had a large proportion of peaks unique to our multi-mapping analysis (supplementary fig. S6, Supplementary Material online). Using this new data set, we observed greater enrichment of H3K27ac at the ancestral *DUSP22* versus *DUSP22B*, which we verified at three PSVs using ChIP-qPCR (1.1–2.9-fold difference of ChIP signal; 1.1–2.9-fold difference of dC_t_ values) (supplementary fig. S7*A*, Supplementary Material online). We also noted a correlation of *DUSP22*/*DUSP22B* expression divergence (Pickrell et al. 2010) (supplementary table S4, Supplementary Material online) and differential H2K27ac enrichment at two of these PSVs (supplementary fig. S7*B*, Supplementary Material online). These findings indicate that reanalysis of ChIP-seq data can accurately identify enriched regions at HSD loci, uncovering potentially divergent regulatory environments.

### Improved Peak Discovery Using Longer-Read ChIP-Seq

To improve our ability to align reads accurately to specific paralogs, we generated longer-read (∼500 bp insert size, 2 × 250 bp PE Illumina) ChIP-seq (“long ChIP”) libraries (H3K4me3, H3K27ac, H3K4me1, and RNA PolII) from the LCL GM12878. Longer reads mapped to SDs with greater accuracy (supplementary fig. S8*A*, Supplementary Material online), allowing for higher-confidence discovery of novel peaks in duplicated regions using standard single-site mapping approaches. However, all marks except H3K4me1 were still depleted for peaks in SDs relative to the rest of the genome. Subsequently, we analyzed the long ChIP data allowing for multiple alignments and probabilistically assigned reads to one position (Bowtie and CSEM, fig. 3*A* and supplementary fig. S8*B*, Supplementary Material online). Long ChIP showed increased posterior assignment probabilities with respect to the short-read ENCODE data (supplementary fig. S8*B*, Supplementary Material online), and the depletion of peaks in SDs was erased for H3K4me3, H3K4me1, and PolII (fig. 3*A*, in pink). Notably, for most libraries, fewer overall peaks were identified with long ChIP versus ENCODE data, though the peaks that did exist were largely replicated (on average, 73% of long ChIP peaks corresponded to ENCODE peaks (Chikina and Troyanskaya 2012); supplementary fig. S9, Supplementary Material online). Long ChIP peaks tended to be larger (2.4–3.7 times as many bases per peak), except for H3K4me1, which had slightly smaller peaks.

### Identification of cCREs

To identify putatively functional *cis*-regulatory regions within HSDs, we integrated our reanalyzed ENCODE and long ChIP data into two 8-state chromHMM models (Ernst and Kellis 2012), from which we identified active promoter- and enhancer-like states that we considered to be cCREs (supplementary fig. S10, Supplementary Material online). This generated a novel set of cCREs in SDs, as virtually no information is available in the current ENCODE release for these loci (fig. 3*B*). Because derived gene expression is broadly lower than ancestral, we quantified the proportion of cCREs covering HSDs in 100-kb windows and observed no significant differences between ancestral and derived loci (defined in Dennis 2017) (Wilcoxon rank-sum test; supplementary fig. S11*A* and *B*, Supplementary Material online). We also observed no differences in the fraction of bases covered between ancestral and derived regions in individual ChIP-seq data sets: H3K27ac, H3K4me3, H3K27ac (data not shown), and heterochromatic H3K27me3 domains (supplementary fig. S11*C*, Supplementary Material online; see Materials and Methods). Thus, explanations beyond the overall abundance of chromatin features are needed, as important functional changes in CRE activity may not be reflected in global differences. For instance, we found that HSD genes whose transcription start site overlapped a cCRE, H3K4me3 peak, or H3K27ac peak had significantly higher expression than those that did not, while the presence of H3K27me3 domains showed the opposite effect (*P *<* *0.05, Wilcoxon rank-sum test) (supplementary fig. S12, Supplementary Material online). We also examined 5′-truncated paralogs, which have lost their ancestral promoters. The transcription start sites of the expressed genes *GTF2IP1* and *GTF2IP4* lie outside of the duplication block and overlapped active promoters (long ChIP cCREs). The next-highest expressed 5′ truncations also show some evidence of active promoters; for example, the *NAIP-B* transcription start site is paralogous to an internal exon of the ancestral *NAIP* and overlaps an H3K4me3 peak not found on *NAIP* (ENCODE multimapping). Overall, we identified differences in the presence or absence of cCREs at paralogous loci (fig. 3*B*), suggesting a more nuanced approach is necessary in pinpointing mechanisms contributing to paralogous expression differences.

### Impact of Nonduplicated Regions on HSD Gene Regulation

**Fig. 4.**
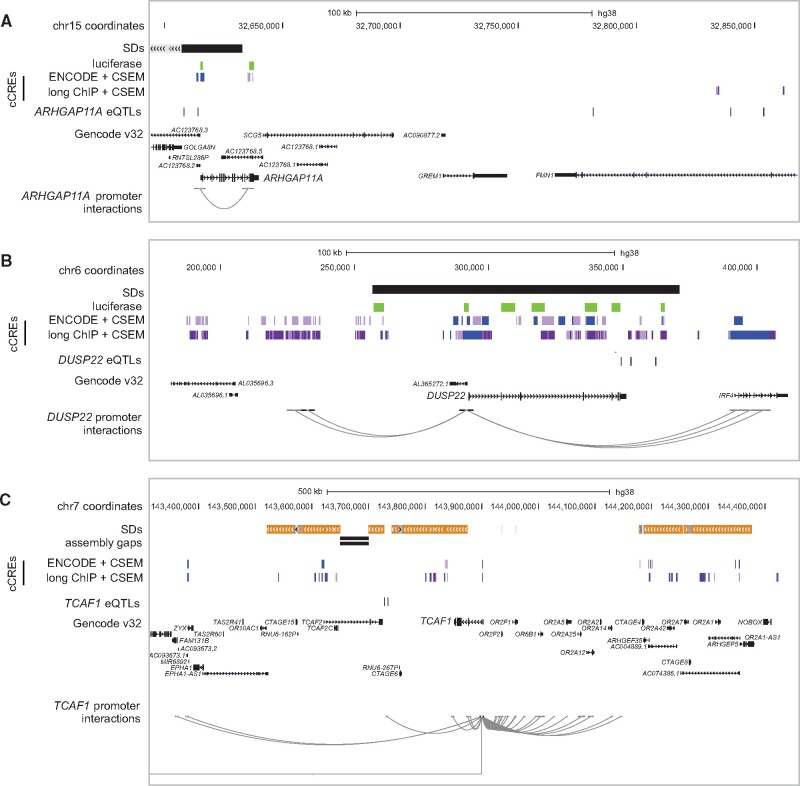
HSD gene regulation in adjacent, nonduplicated regions. Additional regulatory features were examined in the vicinity of the HSD loci, including (*A*) *ARHGAP11A* at chromosome 15q13.1, (*B*) *DUSP22* at chromosome 6p25.3, and (*C*) *TCAF1* at chromosome 7q35. In each panel, SDs are depicted as gray (>90% identical), orange (>98% identical), and black (unannotated) bars. cCREs as defined in this publication are shown in light and dark purple (active enhancer states 1 and 2) and blue (active transcription start site), with luciferase-tested regions in green. eQTLs defined in this publication and regions previously found to interact with HSD promoters are shown for focal genes. Data were visualized in the UCSC Genome Browser (GRCh38).

HSDs are often transposed many thousands of kilobases from their ancestral loci, and in some cases to different chromosomes. As such, we sought to understand if cCREs outside of our duplicated regions might contribute to paralog-specific regulatory patterns. To do this, we considered physical contacts generated by chromatin looping of HSD promoters with cCREs outside of HSD regions. Using loops identified in GM12878 from promoter capture Hi-C (Mifsud et al. 2015) and H3K27ac HiChIP (Mumbach et al. 2017; Juric et al. 2019), we identified 352 and 26 promoter-interacting regions, respectively (mean size ∼5 kb). We found 59 ENCODE multimapping and 106 long ChIP cCREs interacting with an HSD gene promoter. For instance, a chromatin loop connects the *ARHGAP11A* promoter with a cCRE overlapping its nonduplicated 3′-UTR (fig. 4*A*). The majority (>90%) of promoter-interacting regions reside outside of HSDs, in part due to limitations of Hi-C analysis across duplicated loci (Zheng et al. 2019) (see supplementary Note, Supplementary Material online). These findings indicate that proximal nonduplicated regions may play a role in regulating duplicated genes.

We next performed expression quantitative trait locus (eQTL) mapping of HSD genes using our reanalyzed RNA-seq data and existing variant calls from the 1000 Genomes Project (*N* = 460) (1000 Genomes Project Consortium et al. 2015). From this, we identified 40 HSD genes with significant eQTLs, an increase of 1.5- to 4-fold from published work (Lappalainen et al. 2013; Wen et al. 2015). These eQTLs consisted of 3,279 variants in 8,774 gene-variant pairs. A majority (68%) of eQTLs were located within annotated SDs, but variants identified within SDs are often unreliable (Hartasánchez et al. 2018; Ebbert et al. 2019). Accordingly, we focused on the 1,049 eQTLs in SD-proximal non-duplicated regions and found 439 of them had single-gene associations. For example, four variants were associated with *ARHGAP11A* expression (fig. 4*A*), while none were identified for *ARHGAP11B* located ∼2 Mb proximal to its ancestral locus. Similarly, four eQTLs were identified for *DUSP22* on chromosome 6 (fig. 4*B*), though all were located in an SD, while 26 variants were linked with the derived paralog *DUSP22B* on chromosome 16. We intersected SD-proximal eQTLs with our cCREs, reasoning that functional elements would be sensitive to genetic variation and, thus, contain eQTLs. We found that five ENCODE multimapping and 15 long ChIP cCREs contained an HSD eQTL. Finally, 169 eQTLs fell within loci showing significant Hi-C interactions with HSD promoters (31 of these regions, total size ∼160 kb). For instance, the *TCAF1* promoter interacts with a region ∼170 kb downstream that is near two SNPs associated with *TCAF1* and *TCAF2* expression (fig. 4*C*). Altogether, these findings highlight the potential for adjacent, unique sequences to drive divergent regulation of HSDs genes.

### Differential Activity of *Cis*-Acting Elements between Paralogs

**Fig. 5.**
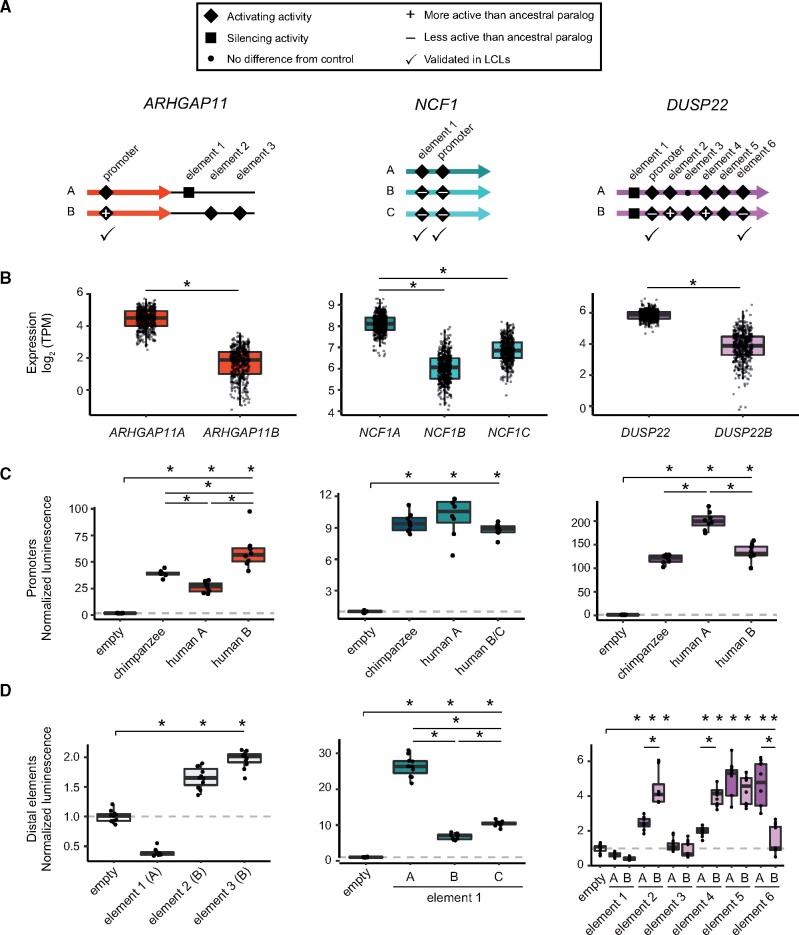
Functional characterization of cCREs in HSDs. cCREs (putative promoters and enhancers) from three HSD duplicate gene families (*ARHGAP11*, *NCF1*, and *DUSP22*) were tested in luciferase reporter assays for activity. (*A*) Cartoons indicating the relative locations of each candidate sequence within or adjacent to HSDs (thick, colored arrows). All experiments (supplementary figs. S16 and S17, Supplementary Material online) are summarized as follows: inactive sequences are shown with a small dot, activating sequences are shown with a diamond, and silencing sequences are shown with a square; differentially active derived sequences (relative to ancestral) are marked with a plus or minus sign; elements tested (and validated) in LCLs are indicated with a check mark. (*B*) mRNA levels (TPM) for the three tested HSD gene families in human LCLs (*N* = 445). (*C*) Representative luciferase reporter experiments for promoters of the paralogous HSD genes and orthologous chimpanzee sequences in HeLa cells. Significantly different activity (*P *<* *0.05, Tukey’s test following ANOVA) from the negative control is indicated along the top bar over each panel, and significant differences among homologous sequences are indicated between boxplots. The *P*-values for each comparison for all experiments are available in supplementary table S7, Supplementary Material online. (*D*) Representative luciferase reporter experiments for candidate enhancers from the same gene families in HeLa cells, with significant activity over/under baseline indicated along the top bar, and significant differences between paralogous sequences between boxplots (*P *<* *0.05, Tukey’s test following ANOVA).

Using our combined data sets, we examined three HSD loci containing gene families expressed highly in LCLs (*ARHGAP11, NCF1*, and *DUSP22*) to identify functional changes in CREs that may contribute to paralogous expression divergence (fig. 5*A*, supplementary figs. S13–S15, Supplementary Material online). In all three cases, the ancestral paralog exhibited significantly greater expression compared to derived paralog(s) (fig. 5*B*). To determine if sequence differences within CREs identified from our chromHMM annotations were sufficient to drive differences in gene expression, we performed luciferase reporter assays on paralogous promoters and enhancer candidates in HeLa cells and LCLs.

#### *ARHGAP11A*/*ARHGAP11B*

The promoter of *ARHGAP11B* exhibited greater activity compared to the chimpanzee ortholog and ancestral paralog in both HeLa and LCLs (∼4-fold difference in activity between HSD paralogs, *P *<* *5 × 10^−10^ in both cell lines; fig. 5*C*, supplementary figs. S16*A* and S17*A*, Supplementary Material online). This was in contrast to mRNA levels in LCLs, where the ancestral *ARHGAP11A* was more highly expressed (fig. 5*B*). With no CREs identified within the shared *ARHGAP11* HSD, we posited that distal elements may drive differential expression between these paralogs. We identified putative enhancers unique to each paralog outside of the shared HSD, which comprised one downstream of the *ARHGAP11A* duplicon (that was also found to interact with the promoter from our Hi-C analysis) and two downstream of *ARHGAP11B*. In HeLa cells, the *ARHGAP11A* element showed weak silencing activity (0.3-fold difference, *P *<* *2 × 10^−16^), while the *ARHGAP11B* elements showed modest activity over baseline (∼2-fold difference, *P *<* *2 × 10^−14^ each), leaving the primary driver of differential expression for these genes undetermined (fig. 5*D*, supplementary fig. S16*B*, Supplementary Material online). While these results were discordant with the mRNA expression of *ARHGAP11* paralogs in LCLs, they may help to explain the unique expression of *ARHGAP11B* in other cell types, such as cortical progenitor neurons (Florio et al. 2015).

#### *NCF1*/*NCF1B*/*NCF1C*

Promoters of the ancestral *NCF1* and its derived paralogs *NCF1B* and *NCF1C* genes did not exhibit significant differential activities in LCLs and modest differences in HeLa (0.8-fold difference, *P* < 0.001; (fig. 5*C*), supplementary figs. S16*A* and S17*A*, Supplementary Material online). However, an enhancer element common to all three paralogs showed the greatest activity for the ancestral *NCF1* paralog in both cell types (fig. 5*D*). This was concordant with differential mRNA levels (∼3-fold difference over either derived in LCL; *P *<* *0.002 for all comparisons) (fig. 5*B*, supplementary fig. S17*B*, Supplementary Material online). Thus, this enhancer, if targeted to *NCF1* and its paralogs, may contribute to differences in their mRNA levels.

#### *DUSP22*/*DUSP22B*

*DUSP22* (ancestral) and *DUSP22B* (derived) promoters showed differential activity concordant with their gene expression in both HeLa and LCLs (i.e., the human ancestral paralog exhibited significantly greater activity than both the human derived and chimpanzee ortholog; ∼1.5-fold difference; *P *<* *5 × 10^−13^) (fig. 5*C*, supplementary figs. S16*A* and S17*A*, Supplementary Material online). We also tested six putative enhancers shared between the two paralogs in HeLa cells and found four active elements, of which two showed differential activity opposite to that of gene expression and one tracked with differential paralog expression (fig. 5*D*). We subsequently validated the latter enhancer element in LCLs (∼1.4-fold difference; *P *<* *2 × 10^−16^) (supplementary fig. S17C, Supplementary Material online). From this, we concluded that the difference in promoter activity is the primary driver of *DUSP22* and *DUSP22B* differential expression, though distal CREs also likely play a role in modulating transcription. Taken together, results from our reporter assays demonstrated that small sequence differences in HSDs can alter *cis*-regulatory activity.

### Putative Mechanisms Contributing to Differential Expression

In search of potential *trans* effectors driving differential expression of these HSD genes, we identified transcription factor binding sites within the assayed sequences. The derived *ARHGAP11B* promoter exhibited greater strength in a reporter assay versus the ancestral paralog and chimpanzee ortholog; we noted a single PSV that more than doubles the number of significant motif matches of the more active *ARHGAP11B* promoter for the transcriptional activators FLI1, GABPA, ETS1, and ELK1 (supplementary fig. S18, Supplementary Material online). Based on chimpanzee homology, these are likely *ARHGAP11A*-specific losses, which matches its reduced activity relative to the derived paralog and chimpanzee ortholog (fig. 5). Examining predicted binding sites within *NCF1* promoters, which did not exhibit differential activity, we observed no gains or losses of any transcription factor recognition sites relative to chimpanzees. No predicted sites were unique to the most active *NCF1* enhancer, but the paralogous *NCF1B* and *NCF1C* possessed many binding sites that were missing from the ancestral, at least one of which belonged to the transcriptional repressor ZNF394. Finally, a deletion of four bases from a homopolymer repeat in the ancestral *DUSP22* promoter removes 13 similar binding sites found only in the less active *DUSP22B* and chimpanzee *DUSP22* ortholog. Some of these belonged to transcriptional repressors (ZNF394 and ZNF350), consistent with their differential transcription. Overall, these findings provide a plausible mechanism for the divergent regulatory activity of a targeted set of duplicated CREs within HSDs.

## Discussion

In this work, we provide evidence that recently duplicated, human-specific genes exhibit differential expression at least in part due to divergent *cis*-acting regulation. Historically, these regions have been poorly characterized genetically and epigenetically. By comparing expression of human and chimpanzee homologs, we assayed potential mechanisms driving duplicate gene fates at relatively short evolutionary time scales (<6 My). To simplify our comparisons of human and chimpanzee orthologs, we assayed gene families with unique duplications in the human lineage but found at single copies in other great apes. As a consequence, notable human-expanded genes such as *NOTCH2NL* (Fiddes et al. 2018; Suzuki et al. 2018), *AMY1* (Perry et al. 2007), and *TBC1D3* (Ju et al. 2016) were excluded from this study. Focusing on human LCLs, we characterized active chromatin features in HSDs and used these candidates to identify differentially active paralogous CREs. Our assessment failed to identify a universal factor responsible for the observed differential expression between paralogs, indicating the underlying molecular mechanisms are likely unique to each HSD gene. Though this work represents an important step toward a more complete picture of HSD gene regulation, there are still some technical limitations to overcome primarily related to using short-read functional-genomic data to assess nearly identical duplications (see supplementary Note, Supplementary Material online). Accurate long-read sequencing (e.g., PacBio HiFi) alleviates many of these issues and, as these technologies become more affordable and widely used, we will be able to more confidently assay mechanisms of gene regulation at HSD loci.

In agreement with previous analyses of whole-genome duplications in teleost fishes (Sandve et al. 2018) and small-scale duplications in yeast (Gu et al. 2005), we found evidence for asymmetric conservation of duplicate gene expression. Specifically, human derived paralogs showed reduced and more divergent expression, recapitulating results in *Drosophila* (Assis and Bachtrog 2013). We suggest this is because derived HSD genes may not be redundant if the full coding sequence or ancestral regulatory environment is missing, resulting in relaxed selection facilitating pseudogenization or neofunctionalization. This is likely for HSDs, which are interspersed throughout the human genome hundreds to thousands of kilobases from each other. As such, daughter paralogs may have been immediately removed from ancestral CREs or placed in novel regulatory environments, such as topological domains, heterochromatin, or transcriptional hubs, causing derived HSD genes to acquire new expression patterns at “birth.” This is particularly likely for 5′-truncated genes. Accordingly, even very recent (<1 My ago) duplications (Dennis et al. 2017), such as gene families *DUSP22*, *SERF1*, *SMN*, *TCAF1*, and *TCAF2*, exhibited differential expression between paralogs.

The young age of HSDs may also explain the lack of subfunctionalization observed in these data; while subfunctionalization is suggested to favor duplicate retention in the long term (Rastogi and Liberles 2005), many HSD genes are less than 2 My old, and partitioning of expression is not expected to arise this quickly (Force et al. 1999). Lan and Pritchard (2016) concluded that in mammals neo- and subfunctionalization evolve slowly and are favored with greater genomic separation, especially for paralogs on different chromosomes. While their study discarded many of the HSD genes highlighted here, due to high sequence identity or classification as pseudogenes, our results are broadly in agreement. Meanwhile, our lack of evidence for dosage sharing as a common outcome in HSDs stands in contrast to that of Qian et al. (2010), who reported an inverse relationship between expression and number of paralogs in duplicates arising since the split of the human and mouse lineages, as well as the ancient split of the fission and budding yeasts (>300 My). However, HSD genes are over an order of magnitude younger, providing a novel glimpse at gene regulation in very recent duplicates, many of which may not be retained. We again suggest that while expression changes reported here apparently arose rapidly, dosage compensation or subfunctionalization in general may take longer to evolve. Importantly, subfunctionalization and neofunctionalization are not mutually exclusive processes, and more stringent criteria defining subfunctionalization can make it harder to identify (Huminiecki and Wolfe 2004). Finally, while we cannot discount increased dosage of functionally redundant paralogs within a gene family as contributing to unique human traits (fig. 1*D*), we note that a little over half of our HSD genes represent partial duplications with likely altered protein functions, as observed for *SRGAP2C* (Charrier et al. 2012; Dennis et al. 2012) and *ARHGAP11B* (Florio et al. 2015). Thus, additive dosage effects must be considered for each gene on a case-by-case basis.

Our expression data offer some insights into the functions of previously uncharacterized HSD genes. Though our primary analysis used LCLs, a cell type not commonly associated with human-specific features such as altered brain and musculoskeletal morphology, there is evidence of immune-related differences across great apes (Barreiro et al. 2010). Further, it has been suggested that humans are more prone to autoimmune diseases than chimpanzees, particularly as a result of T- and B-cell response to viral infection (Jimenez and Piera-Velasquez 2013; Varki 2017). In our expression comparisons of chimpanzee and human orthologs, *ARHGEF35* stood out as a potentially neofunctionalized gene, as it exhibited lower cross-tissue correlation with chimpanzee, higher tissue specificity, yet globally higher expression in multiple human tissues versus its ancestral paralog *ARHGEF5* (supplementary fig. S2, Supplementary Material online; supplementary table S3, Supplementary Material online). Though little is known about its function, *ARHGEF35* encodes a truncated version of *ARHGEF5*, a Rho guanine nucleotide exchange factor capable of activating Rho-family GTPases (Rossman et al. 2005) that plays a role in inflammatory response and dendritic cell migration (Wang et al. 2009). We also speculate that two of our highlighted genes—*NCF1*, encoding Neutrophil Cytosolic Factor 1, and *DUSP22*, encoding a tyrosine phosphatase—may contribute to variation in protection against autoimmune response mediated by gene dosage. *NCF1* knockout causes increased T-cell activity in mice, resulting in arthritis and encephalomyelitis phenotypes (Hultqvist et al. 2004). While derived paralogs *NCF1B* and *NCF1C* are rendered nonfunctional in humans due to a frameshift mutation, in some individuals they encode the ancestral sequence as a result of interlocus gene conversion (Heyworth et al. 2002). Because increased *NCF1* CN is associated with reduced risk of systemic lupus erythematosus (Zhao et al. 2017), gene conversion of the derived paralogs could act to maintain redundant, functional sequence variants (Teshima and Innan 2008) with an advantageous additive effect. *DUSP22* also regulates immune response, with knockout mice exhibiting enhanced T-cell proliferation, increased inflammation, and autoimmune encephalomyelitis (Li et al. 2014). The full-length paralog *DUSP22B* is located on chromosome 16p12.1 at variable CN but is functionally uncharacterized and missing from the human reference. No gene-disrupting mutations were identified for either paralog in hundreds of population controls (Dennis 2017), making it plausible that *DUSP22B*, which is expressed at variable dosage in humans (fig. 2*B*), is functionally redundant with *DUSP22* and could similarly play a protective role in autoimmunity. While only a proxy for function, our analysis of HSD gene expression is helpful in prioritizing genes for future assessments.

To better understand how altered CREs may contribute to paralogous expression divergence, we experimentally dissected three HSD gene families and found promoter activity was only sometimes concordant with overall gene expression, suggesting that other types of regulatory elements, like enhancers and silencers, may cooperatively control overall expression. Currently, the challenge is to pinpoint functional CREs impacted by PSVs or residing within nonduplicated regions that may differentially alter specific paralogs. We have produced and leveraged a variety of analyses to narrow down likely candidates by chromatin state, expression modulation, and physical proximity to promoters. However, the number of candidate regions is too great to test via low-throughput methods such as luciferase reporter assays. This problem is exacerbated by the need to compare regulatory behavior across multiple cell types. To address this, massively parallel reporter assays should be employed to validate and quantify CRE activity of thousands of candidate paralogous sequences. Such data could determine to what extent HSD gene expression is predicted by nearby regulatory regions. We could also integrate additional types of data, such as targeted chromatin capture of CREs within SDs (such as capture Hi-C) or nascent transcription (GROseq, 5′ CAGE). Finally, characterization of DNA methylation, which is especially challenging in duplicated loci, will be vital to build a more complete picture of the epigenetic landscape. This study represents a first step toward improving quantification of gene expression and active chromatin states in recent duplications and provides a foundation for future work characterizing regulatory and functional changes in recently duplicated loci.

## Materials and Methods

### Quantification of HSD Gene Expression

Iso-Seq filtered alignments were obtained from the ENCODE portal (Davis et al. 2018). Reads were counted per HSD gene with HTSeq (Anders et al. 2015) before calculating reads per kilobase of transcript, per million mapped reads values. For supplementary figure S3*B*, Supplementary Material online *DUSP22* and *DUSP22B* reads were counted separately based on PSV-containing sequence using SAMtools mpileup. Human and chimpanzee RNA-seq data were quantified alignment-free with custom reference transcriptomes. Expression quantification was performed using Salmon v1.2.0 (Patro et al. 2017), the custom transcriptomes, and reference genomes (GRCh38 or from Kronenberg et al. (2018) for chimpanzee) as a decoy sequence. For paired-end data, we used the flags “–validateMappings” and “–gcBias”. RNA-seq data were first lightly trimmed prior to quantification using trim_galore with the following flags: -q 20 –illumina –phred33 –length 20. Length-normalized TPM values or counts per gene were obtained using the tximport package in R (Soneson et al. 2015). See supplementary Materials and Methods, Supplementary Material online for more detailed descriptions.

### Differential Expression Analysis

Human and chimpanzee RNA-seq data from four primary tissues (Blake et al. 2020), LCLs (Khan et al. 2013; Blake et al. 2020), iPSCs (Pavlovic et al. 2018), and iPSC-derived neural progenitor cells (Marchetto et al. 2019) were analyzed as described above. Count data from chimpanzee genes were duplicated to allow for pairwise comparison to each HSD duplicate, as well as the sum of all HSD genes in each family. Genes expressed below the 75% percentile (corresponding to 1–2 counts per million reads) were filtered from the analysis, leaving 16,752–18,225 genes. A linear model including species and sex was fitted to each shared gene (*N* = 55,461) using limma-voom (Law et al. 2014; Ritchie et al. 2015), and DE genes were identified at a 5% false discovery rate (FDR). For ancestral-derived comparisons in the human LCLs, TPM values were log-transformed using a pseudocount of 1 × 10^−4^ (an order of magnitude below the smallest nonzero value), compared with a Wilcoxon signed-rank test, and considered significant at a 5% FDR (Benjamini–Hochberg).

### CN-Controlled Differential Expression Analysis

Paralog-specific CN estimates were generated using QuicK-mer2 (Shen and Kidd 2020), whole-genome sequence data from the 1000 Genomes Project (30X) (Fairley et al. 2020), and a custom reference consisting of GRCh38 plus an additional contig representing the *DUSP22B* duplicon (Dennis et al. 2017). Expression analysis was performed using RNA-seq data from LCLs included in the Geuvadis study (Lappalainen et al. 2013) for which CN genotypes were generated (*N* = 445). Ancestral-derived gene pairs were compared with a linear model to identify significant differences in log_2_-transformed TPM values after controlling for continuous CN genotypes. Models were first fit with an interaction coefficient, and if no interaction was detected (*P *>* *0.05), models were fit to expression and CN only. Resulting *P*-values were corrected via the Benjamini–Hochberg procedure using the R package qvalue (http://github.com/jdstorey/qvalue) and used to identify differential expression of ancestral-derived gene pairs at a 5% FDR. For visualization purposes (fig. 2*B*), *DUSP22* CN genotypes were adjusted to known absolute values for GM12878 (as determined by fluorescence *in situ* hybridization in Dennis et al. (2017)).

### Identification of miRNA Binding Sites

For ancestral paralogs of each HSD gene family, the 3′-UTR was extracted from canonical transcript isoforms using the UCSC Genome Browser (GRCh38) and compared using blastn (Altschul et al. 1990) against existing alignments of homologs previously generated for human, chimpanzee, and rhesus (Dennis et al. 2017). Using TargetScan 7.0 and annotated miRNA sequences and families (release 7.1; September 2016) (Agarwal et al. 2015), we identified miRNA targets of individual human paralogs and nonhuman primate orthologs.

### Correlation of Expression Divergence and Sequence Divergence

Ancestral-derived paralog expression divergence was calculated as the absolute value of log_2_(derived/ancestral), using the median TPM values for each gene and a pseudocount 1 × 10^−4^. Sequence divergence as the pairwise identity with the ancestral sequence was taken from Dennis et al. (2017). Gene families were included if at least one paralog was expressed at a level >1 TPM. For promoters, sequence divergence was tabulated as the sum of all mismatches and alignment gaps within ±500 base pairs of the transcription start site (Gencode v32). These quantities were correlated and the strength of the relationship was determined with a linear regression.

### Cell Culture

Human LCLs were obtained from the Coriell Institute. The cells were grown in suspension in RPMI 1640 medium (Genesee Scientific) supplemented with 15% fetal bovine serum, 100 U/ml penicillin, and 100 µg/ml streptomycin and maintained at 37°C with 5% CO_2_. To test the impact of NMD inhibition, two million cells of each LCL (GM19204, GM18508, GM19193, GM19238, GM12878, and S003659) were grown overnight and subsequently treated with 100 µg/ml of emetine (Sigma) for 7 h (Noensie and Dietz 2001). Parallel cultures were left untreated and grown at standard conditions. HeLa cells were grown in Dulbecco's Modified Eagle Medium, High Glucose, with L-Glutamine (Genesee Scientific) supplemented with 10% fetal bovine serum (Gibco, Life Technologies), penicillin (100 U/ml), and streptomycin (100 µg/ml) (Gibco, Life Technologies) at 37°C with 5% CO_2_.

### RNA Extraction and cDNA Generation

LCLs were harvested and added to an appropriate volume of TRIzol^®^ solution (Invitrogen™) (1 ml per 10^7^ cells) and stored at −80°C for ∼24 h before extraction to ensure complete lysis of cells. The next day, 200 μl of chloroform (Fisher Scientific) was added, and the homogenate was shaken vigorously for 20 s and incubated at room temperature for 2–3 min. Samples were spun at 10,000×*g* for 18 min at 4°C and the aqueous phase was transferred to a sterile RNase-free tube. An equal volume of 100% RNAse-free ethanol was added, samples were mixed by vortex, and then purified with an RNeasy Mini Kit (Qiagen). Samples were eluted in 30 μl RNase-free water and stored at -80°C. Transcriptor High Fidelity cDNA Synthesis Kit (Roche) was used for cDNA synthesis with OligodT primers. Following reverse transcription, samples were treated with RNase A (Qiagen) at 37°C, and cDNAs were stored at −20°C.

### ChIP Assays

ChIP assays were carried out as previously described with minor modifications (O’Geen et al. 2019) (see supplementary Material and Methods, Supplementary Material online). ChIP enrichments were confirmed by qPCR with *ACTB* (positive control) and *HER2* (negative controls) (primers in supplementary table S8, Supplementary Material online). ChIP enrichment was calculated relative to input samples using the dC_t_ method (dC_t_ = C_t_[HER2-ChIP] − C_t_[input]). ChIP-seq libraries were prepared using the KAPA Hyper Prep Kit (Roche).

### Analysis of ChIP-Seq Data

ChIP-seq raw data and peaks obtained with the ENCODE pipeline were directly downloaded from the online portal (Davis et al. 2018) (https://www.encodeproject.org/; last accessed May 7, 2021). All ChIP-seq analyses are available as a TrackHub for the UCSC Genome Browser (https://bioshare.bioinformatics.ucdavis.edu/bioshare/download/cpqqdfge5lfvovq/hsd_noncoding/hub.txt; last accessed May 7, 2021). Our ChIP-seq bioinformatic pipeline is freely available for use in Snakemake format (https://github.com/mydennislab/snake-chipseq; last accessed May 7, 2021), allowing the analysis to be replicated in any cell or tissue type of interest. Briefly, Illumina adapters and low quality bases (Phred score < 20) were trimmed using Trimmomatic (Bolger et al. 2014) and aligned to a custom reference genome (GRCh38 with an added *DUSP22B* contig) using single-end Bowtie (Langmead et al. 2009) configured to allow multiple mappings per read. Paired-end long-ChIP reads were also mapped using paired-end BWA-MEM and filtered by MAPQ ≥ 20. After mapping, PCR duplicates and secondary alignments were removed using Picard MarkDuplicates and SAMtools v1.9, respectively. Bowtie multimapping reads were allocated to their most likely position using CSEM v2.4 (Chung et al. 2011) and a custom script was developed to select the alignment with the highest posterior probability. Peaks were called using MACS2 callpeak (v2.2.6) on default settings using the MACS2 shifting model (Zhang et al. 2008). Sets of peaks were compared between analysis methods using HOMER mergePeaks (parameters: “-d given”) (Heinz et al. 2010) and a unidirectional correlation metric derived from IntervalStats using peaks with an overlap *P*-value below 0.05 (Chikina and Troyanskaya 2012). See supplementary Materials and Methods, Supplementary Material online for more detailed descriptions.

For depletion analyses, SD coordinates were directly downloaded from UCSC Table Browser and HSD coordinates were obtained by filtering alignments with sequence identity over 98% in the fracMatch column, converting them to BED format and merging overlapping entries using bedtools merge. The number of peaks and bases under peaks on each region of interest were obtained with bedtools intersect. To obtain depletion statistics, 1,000 regions of the same size as SD and HSD were randomly sampled from the human genome GRCh38. Empirical *P*-values of depletion tests were calculated as *P* = (*M* + 1)/(*N* + 1), where *M* is the number of iterations less than the observed value and *N* is the number of iterations.

Additionally, mapping quality scores (MAPQ) distributions for H3K27ac were generated following a similar approach as explained before, but using BWA-backtrack and BWA-MEM for short and long ChIP-seq reads respectively, based on read length specifications. PCR duplicates and secondary alignments were removed. Posterior probability distributions for H3K27ac were examined using the output of CSEM after selecting the most likely alignment with the custom script. Entries in unique space were subsampled to 10 million and plots were obtained with the geom_density function in ggplot R package.

### ChromHMM Annotations

We generated ChromHMM (version 1.19) (Ernst and Kellis 2012) models separately for ENCODE short-read data and long ChIP after multimapping and CSEM allocation, using active chromatin histone modifications (H3K4me3, H3K4me1, and H3K27ac). States corresponding to active transcription start sites and active enhancers were identified manually (Ernst and Kellis 2017). In the ENCODE analysis, promoters were assigned to state 1, which corresponded to active transcription start sites, and enhancers were assigned to state 8, which corresponded to active enhancers (supplementary fig. S10*A*, Supplementary Material online). Similarly, in the long ChIP analysis, promoters were assigned to state 3 and active enhancers were assigned to states 6; state 4 was considered to be an additional enhancer state lacking enrichment in H3K4me1 (Ernst and Kellis 2017) (supplementary fig. S10*B*, Supplementary Material online). Together, these sets of elements were defined as cCREs.

### Paralog-Specific Validation of RNA Expression and ChIP Data

Following published protocols (Integrated DNA Technologies), we used the rhAMP assay in 10 µl total reaction volumes to quantify abundance of PSVs (for all assays except *ARHGAP11* expression, the fluorophores FAM=A paralog and VIC=B paralog) as a proxy for paralog-specific expression (RNA) and enrichment (ChIP) (supplementary table S8, Supplementary Material online). We used 10 ng total of RNA converted to cDNA to validate gene expression for duplicated gene families *ARHGAP11*, *ROCK1*, and *DUSP22*. We calculated dCt of cDNA and gDNA as Ct_FAM_-Ct_VIC_ and ddCt as dCt_cDNA_-dCt_gDNA_ from the same cell line. We calculated dCt of the input and ChIP-enriched library as Ct_FAM_-Ct_VIC_ and ddCt as dCt_ChIP_-dCt_input_ from the same cell line. For both expression and ChIP analyses, the ratio of abundance of the B to the A paralog is 2^ddCt^.

### Luciferase Reporter Assays

Expression clones for luciferase assays were generated using reporter constructs pGL3-basic (Promega) for promoters and pE1B (Antonellis et al. 2008) for cCREs. Constructs were cotransfected (ThermoFisher Lipofectamine 3000) in equimolar amounts with 50 ng of the control plasmid pRL-TK (Renilla luciferase) into HeLa cells or electroporated using the Neon Transfection System for LCLs in accordance with previously published work (Tewhey et al. 2018). Luciferase assays were performed with the Dual-Luciferase Reporter Assay System (Promega E1910). Luminescence measurements were performed according to the manufacturer’s instructions using a Tecan Infinite or Tecan Spark plate reader with injectors. See supplementary Materials and Methods, Supplementary Material online for more detailed descriptions.

### Transcription Factors Binding Motifs

Alignments of cloned sequences were scanned for HOmo sapiens COmprehensive MOdel COllection (HOCOMOCO) v11 (Kulakovskiy et al. 2018) transcription factor binding site motifs using FIMO (Grant et al. 2011). HOCOMOCO motifs were limited to transcription factors expressed above 1 TPM in >75% of ENCODE mRNA-seq libraries generated for GM12878 (ENCSR077AZT, ENCLB555AQG, ENCLB555AQH, ENCLB555ANP, ENCLB555ALI, ENCLB555ANM, ENCLB555ANN, ENCLB037ZZZ, ENCLB038ZZZ, ENCLB043ZZZ, ENCLB044ZZZ, ENCLB041ZZZ, ENCLB042ZZZ, ENCLB045ZZZ, ENCLB046ZZZ, ENCLB700LMU, ENCLB150CGC). Significant matches above a 5% FDR were retained for the analysis. Transcription factor binding sites were compared across homologous sequences to identify putative paralog-specific gains and losses of binding sites.

## Supplementary Material

Supplementary data are available at *Molecular Biology and Evolution* online.

## Supplementary Material

msab131_Supplementary_Data
