# Supplementary material for: Diverse Molecular Mechanisms Contribute to Differential Expression of Human Duplicated Genes
Source: Mol Biol Evol. 2021 May 1;38(8):3060–77. doi: 10.1093/molbev/msab131 (PMC8321529; doi:10.1093/molbev/msab131)
Supplement: msab131_Supplementary_Data [file msab131_supplementary_data.zip › SupplementaryMaterials_corrected.pdf]

Supplementary Materials for:

Diverse molecular mechanisms contribute to differential expression of human duplicated genes

Colin J. Shew[1,2], Paulina Carmona-Mora[1,3,4], Daniela C. Soto[1,2], Mira Mastoras[1], Elizabeth Roberts[1], Joseph Rosas[1,5], Dhriti Jagannathan[1], Gulhan Kaya[1], Henriette O'Geen[1], Megan Y. Dennis[1-6,†]

[1]Genome Center, [2]Integrative Genetics and Genomics Graduate Group, [3]MIND Institute, [4]Autism Research Training Program, [5]Postbaccalaureate Research Education Program, [6]Department of Biochemistry & Molecular Medicine, University of California, Davis, CA, USA

Abbreviations

cCRE, candidate *cis*-regulatory element
ChIP, chromatin immunoprecipitation
CN, copy number
CRE, *cis*-regulatory element
DE, differential expression
eQTL, expression quantitative trait locus
FDR, false discovery rate
HSD, human-specific segmental duplications
kb, kilobase pair
LCL, lymphoblastoid cell line
NMD, nonsense-mediated decay
PSV, paralog sequence variant
RPKM, reads per kilobase per million mapped reads
SD, segmental duplication
TPM, transcripts per million
UTR, untranslated region

Supplementary Figures

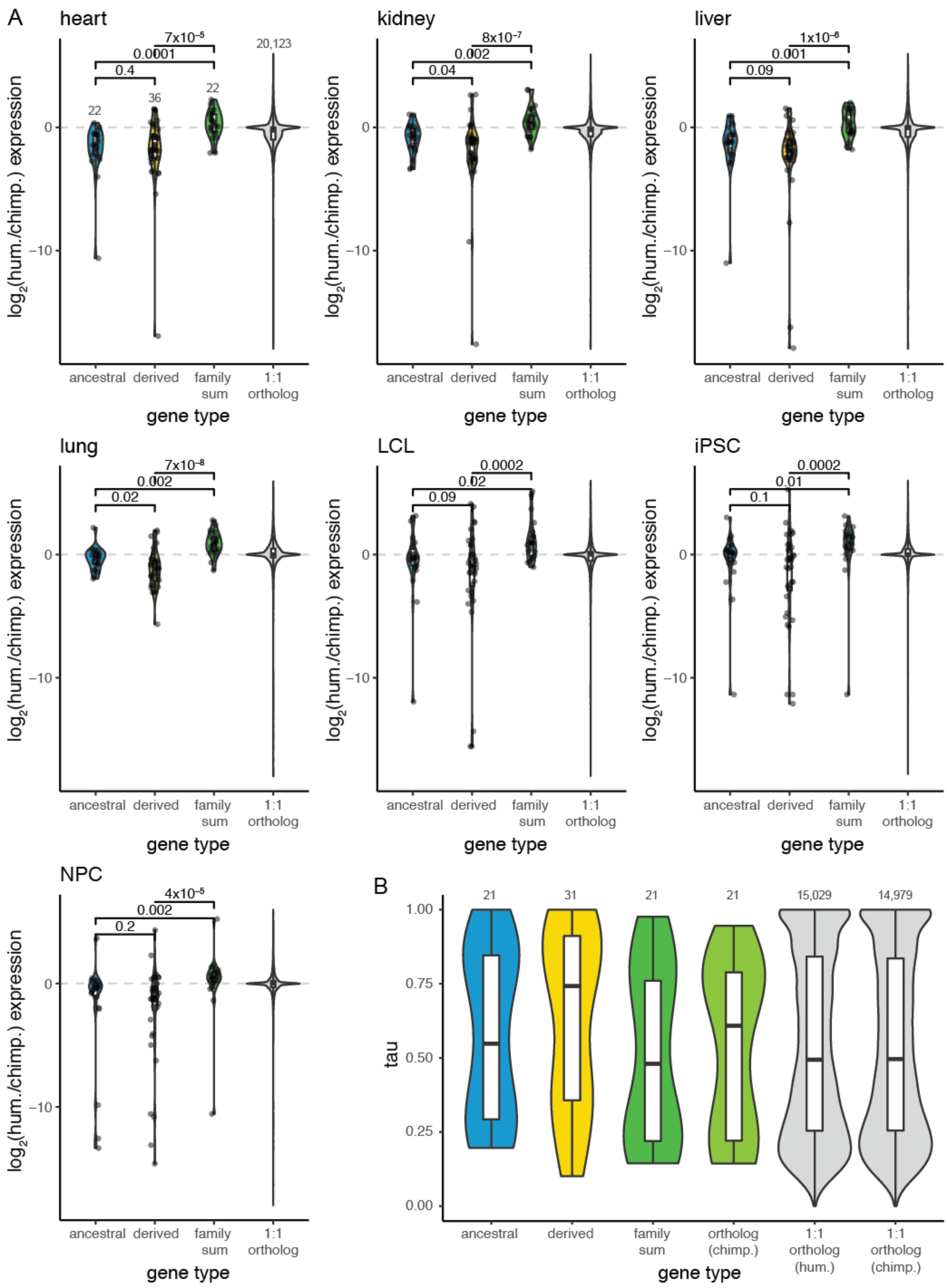

Figure S1. Comparison of human gene expression with chimpanzee orthologs across diverse tissues. Violin and box plots represent relative expression levels (log_2 ratio of human (hum.) versus chimpanzee (chimp.) expression **(A)** and cross-tissue expression correlations (tau) **(B)** across seven tissues/cell types. The pairwise *p*-values indicated above plots are from Dunn's test, following a Kruskal-Wallis test. Differences among tau values were not significant.

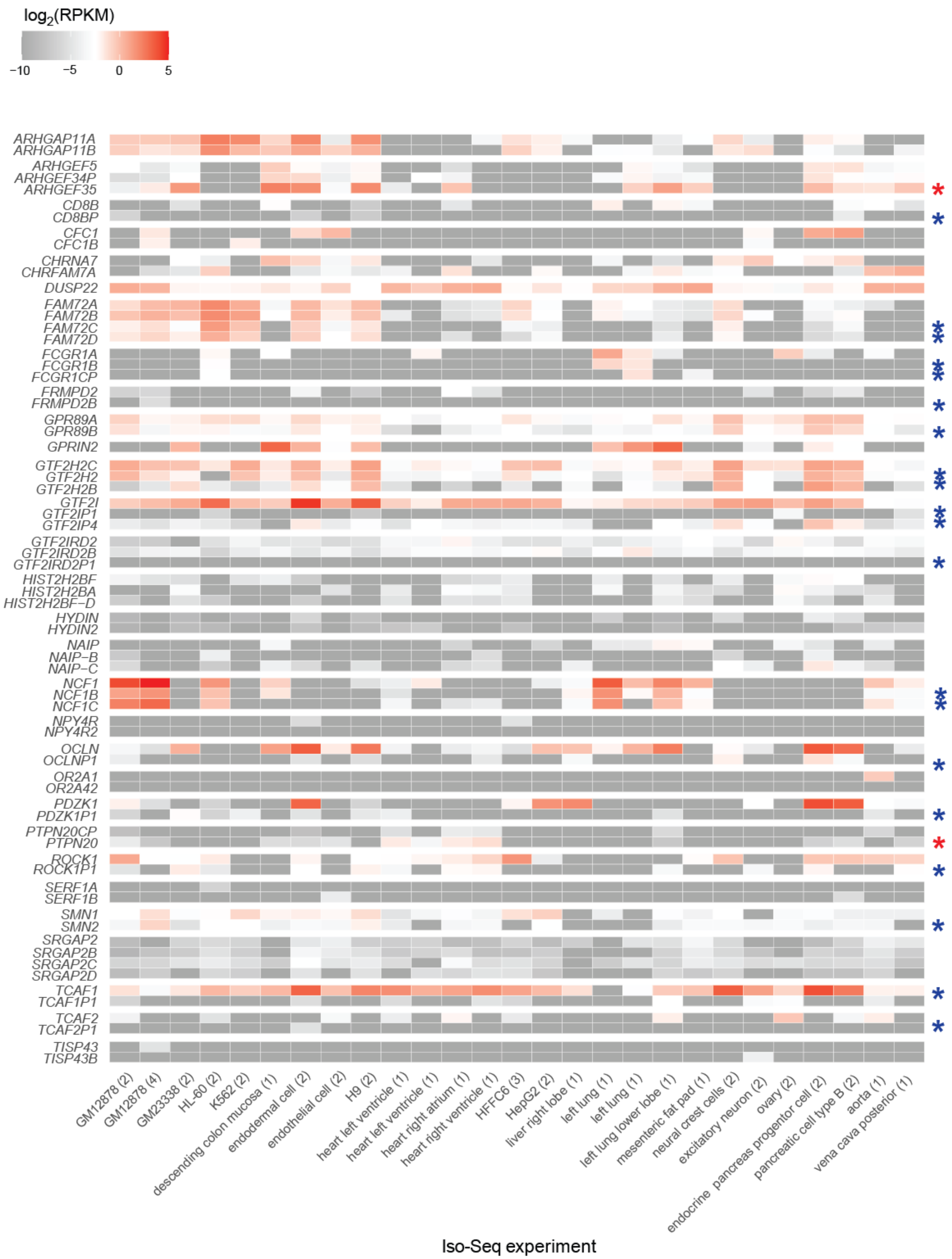

Figure S2. HSD gene expression in Iso-Seq datasets. For each available ENCODE Iso-Seq experiment (columns; number of technical replicates indicated in parentheses), HSD gene expression was calculated in reads per kilobase per million mapped reads (RPKM) using only paralogous regions (i.e., excluding truncated portions and novel portions of genes). For derived genes, $\log_2$(RPKM) values were compared to the ancestral gene with a Wilcoxon signed-rank test. Significant differences (Benjamini-Hochberg adjusted $p < 0.05$) are indicated with an asterisk (blue for lower expression; red for higher). *DUSP22* and *GPRIN2* were not tested for differential expression because their derived genes are missing from GRCh38.

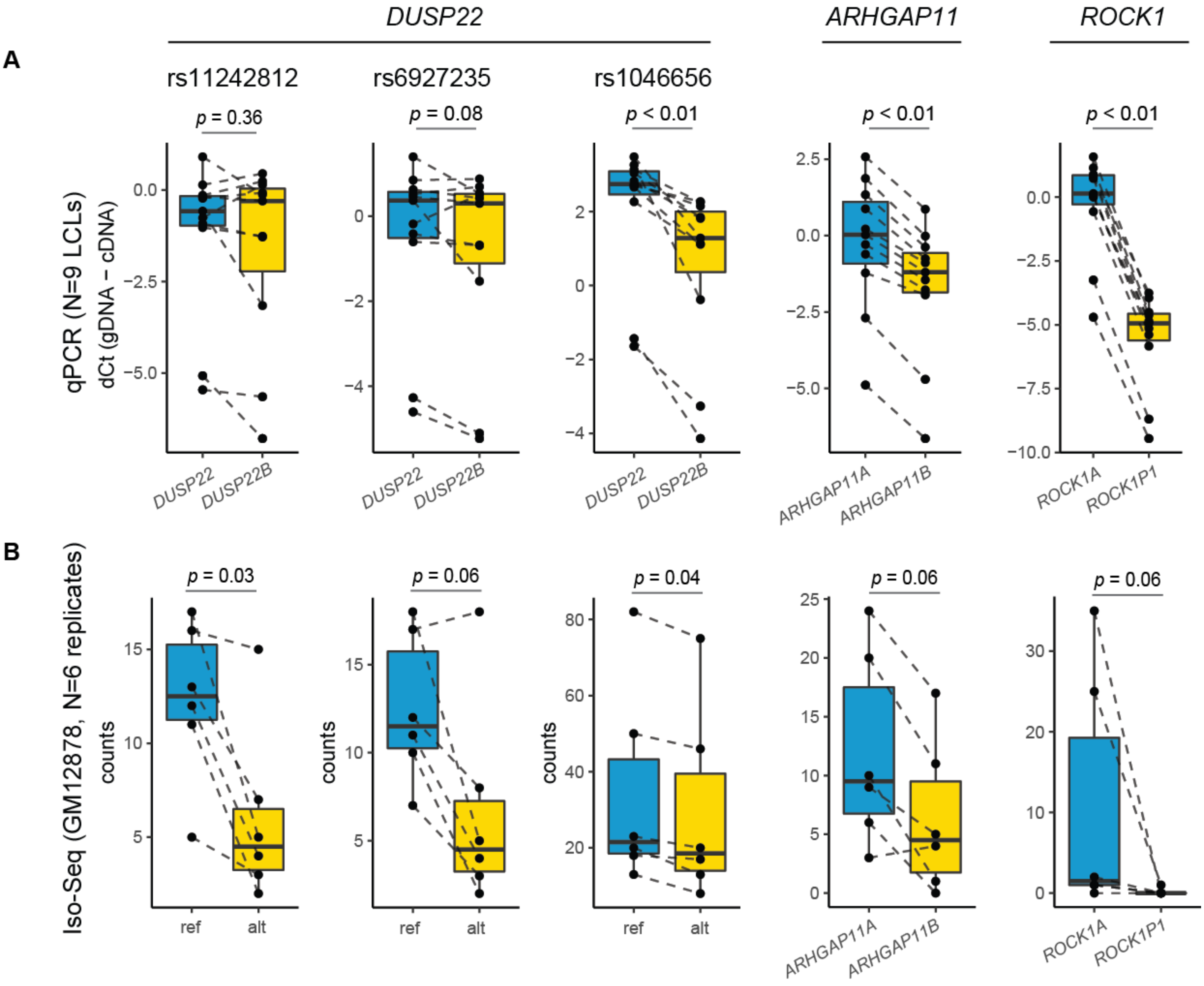

Figure S3. Validation of short-read RNA-seq quantification. (A) qPCR was conducted on genomic DNA (gDNA) and complementary DNA (cDNA) extracted from nine human LCLs to quantify expression differences at paralog-specific variants (PSVs). Because *DUSP22B* is missing from the reference, *DUSP22* PSVs are annotated as SNPs, where the alternate allele corresponds to a known *DUSP22B* substitution. The difference of cycle threshold (dCt) between gDNA and cDNA samples was calculated for each LCL and compared between paralogs with a paired Wilcoxon signed-rank test. Each point represents the mean of three technical replicates. **(B)** Read counts from six replicates of GM12878 Iso-Seq experiments (ENCODE) at *DUSP22* PSVs (raw alignments prior to sequence correction) or *ARHGAP11*/*ROCK1* paralogous regions (filtered alignments). Differences were quantified with a paired Wilcoxon signed-rank test.

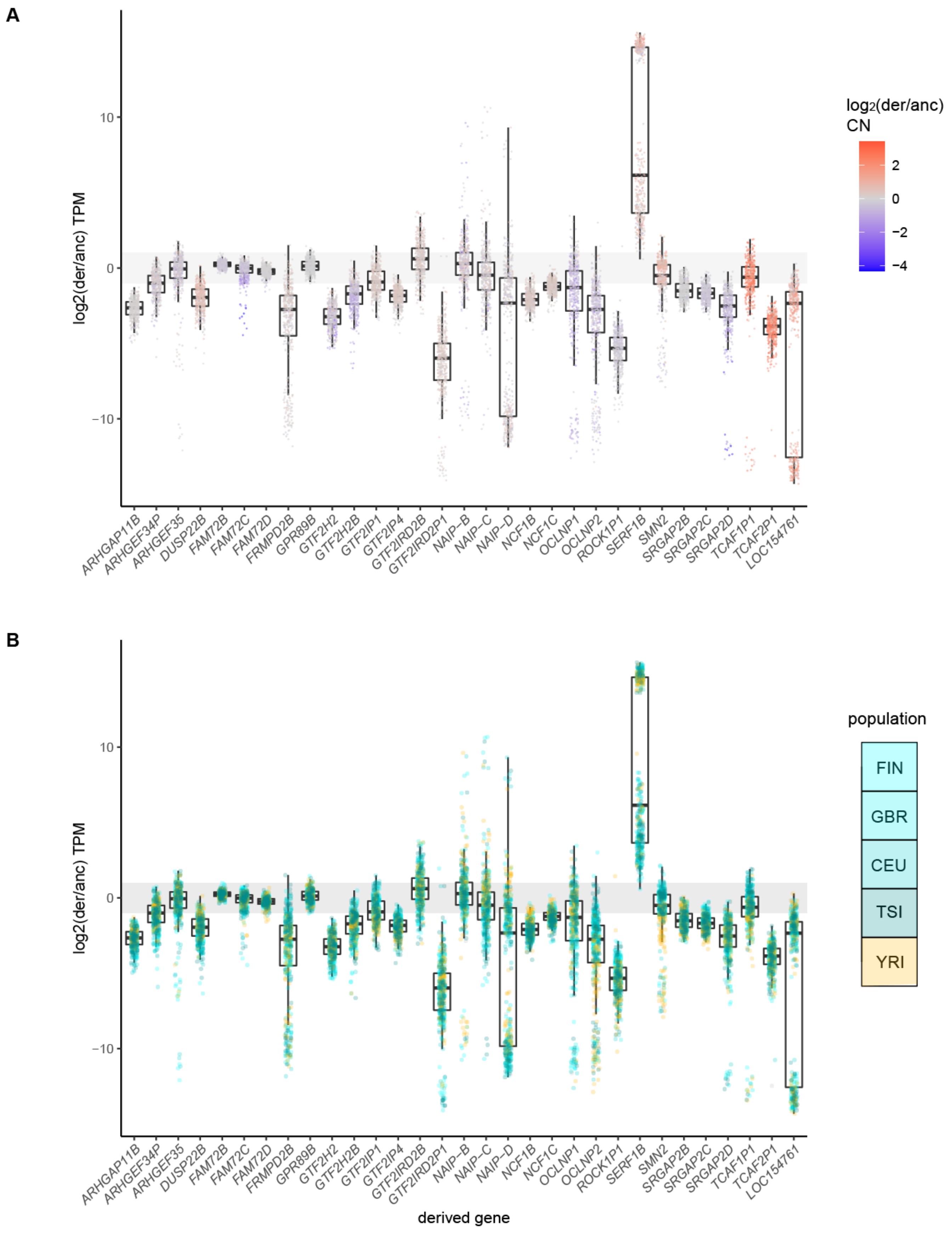

Figure S4. Expression divergence of derived HSD genes. Expression divergence of derived genes from families with at least one LCL-expressed paralog is plotted as the $\log_2$ ratio of median derived and ancestral TPM expression. Each point represents a different LCL from the Geuvadis consortium (total N=445). The gray bar indicates a two-fold expression difference. Colors represent **(A)** relative CN and **(B)** LCL source population.

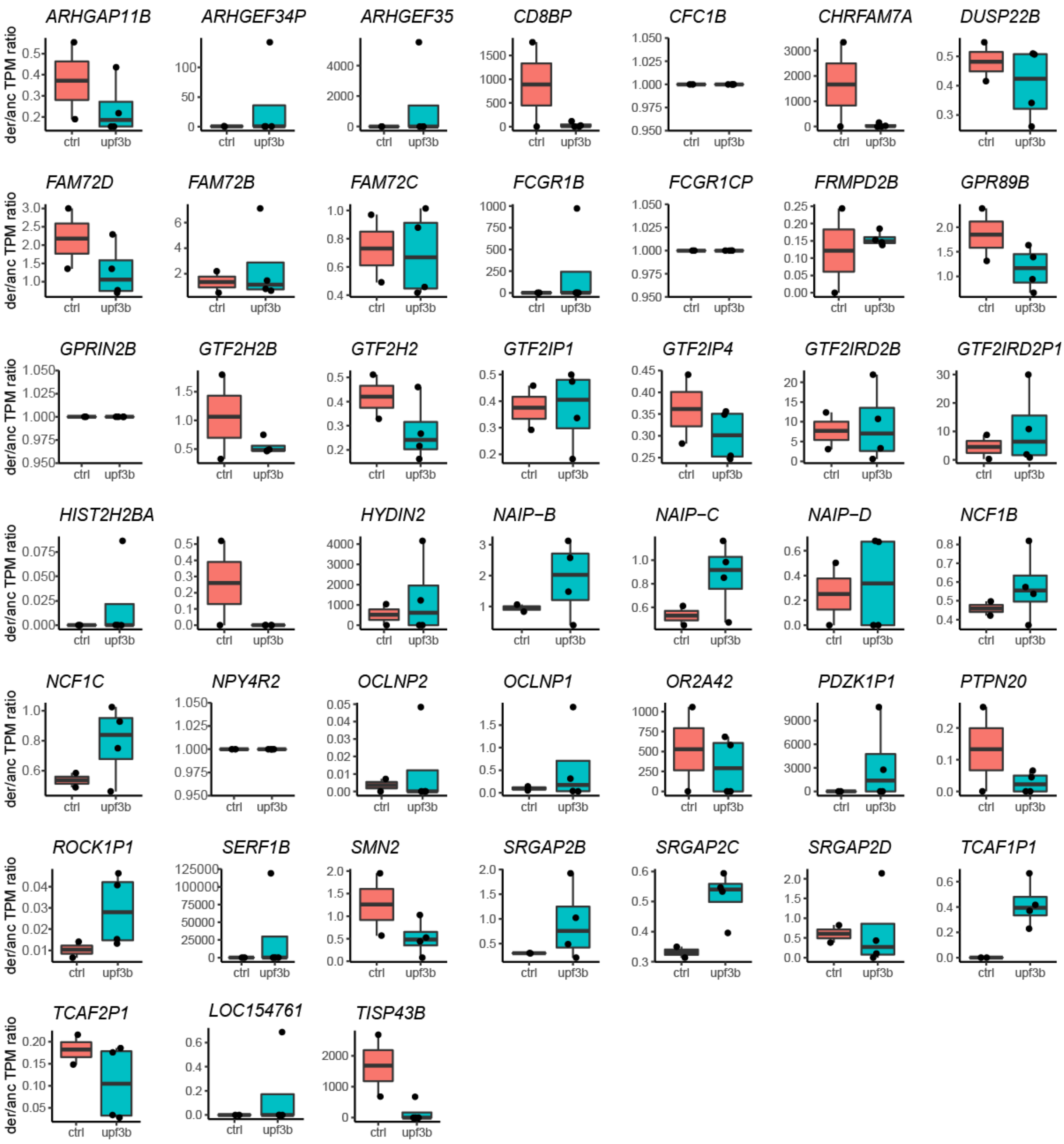

Figure S5. Nonsense-mediated decay of HSD transcripts. Derived/ancestral TPM ratios of HSD genes, calculated from RNA-seq of control (ctrl) and NMD-deficient cells (upf3b) (Nguyen et al. 2012). No differences were determined to be significant by differential expression analysis (limma-voom).

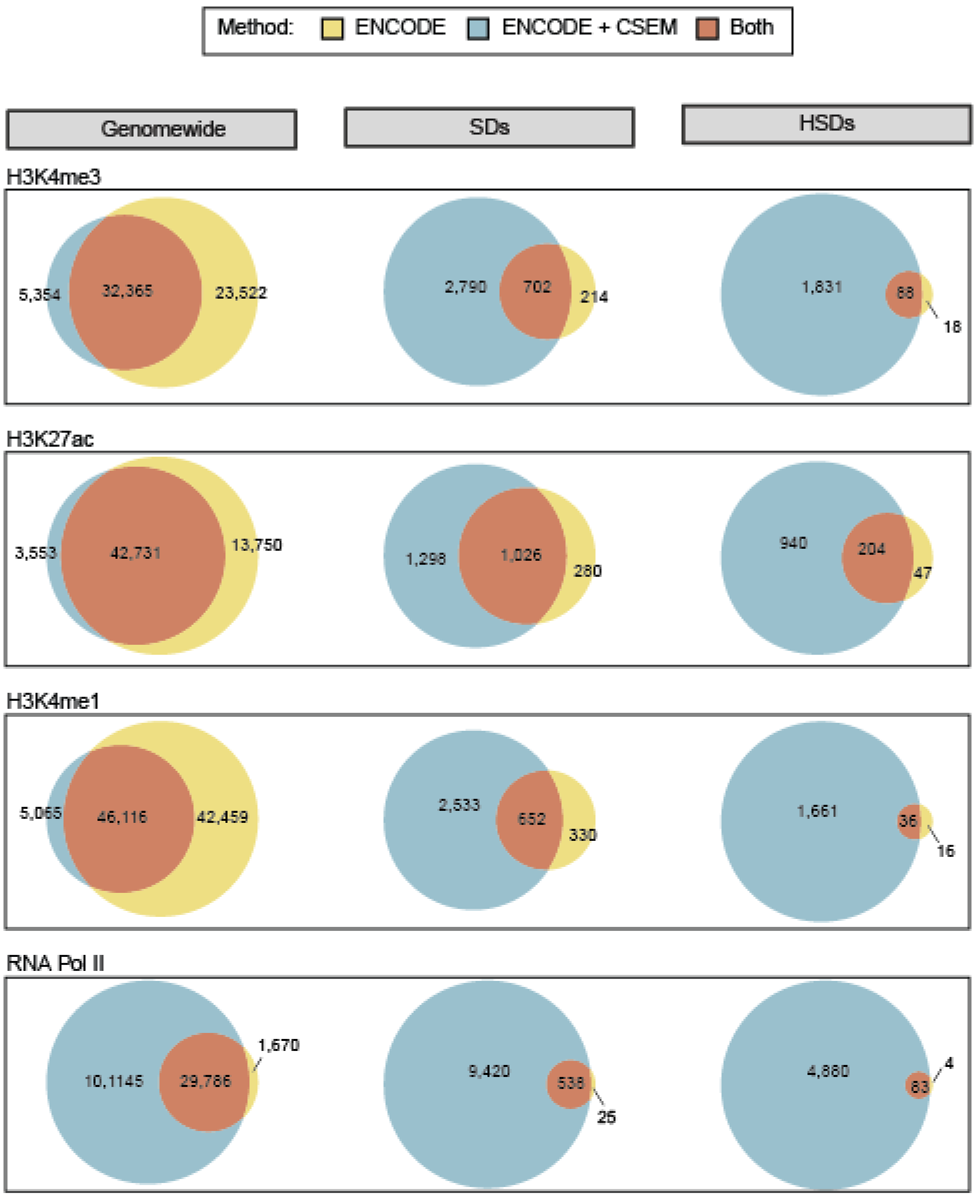

Figure S6. Comparison of ChIP-seq peaks from ENCODE and ENCODE data with multimapping and CSEM allocation. Overlap between data sets is shown for the whole genome, SDs, and HSDs (SDs with over 98% identity) for H3K4me1, H3K27ac, H4K4me3, and RNA PolII. Color indicates the method used for read mapping.

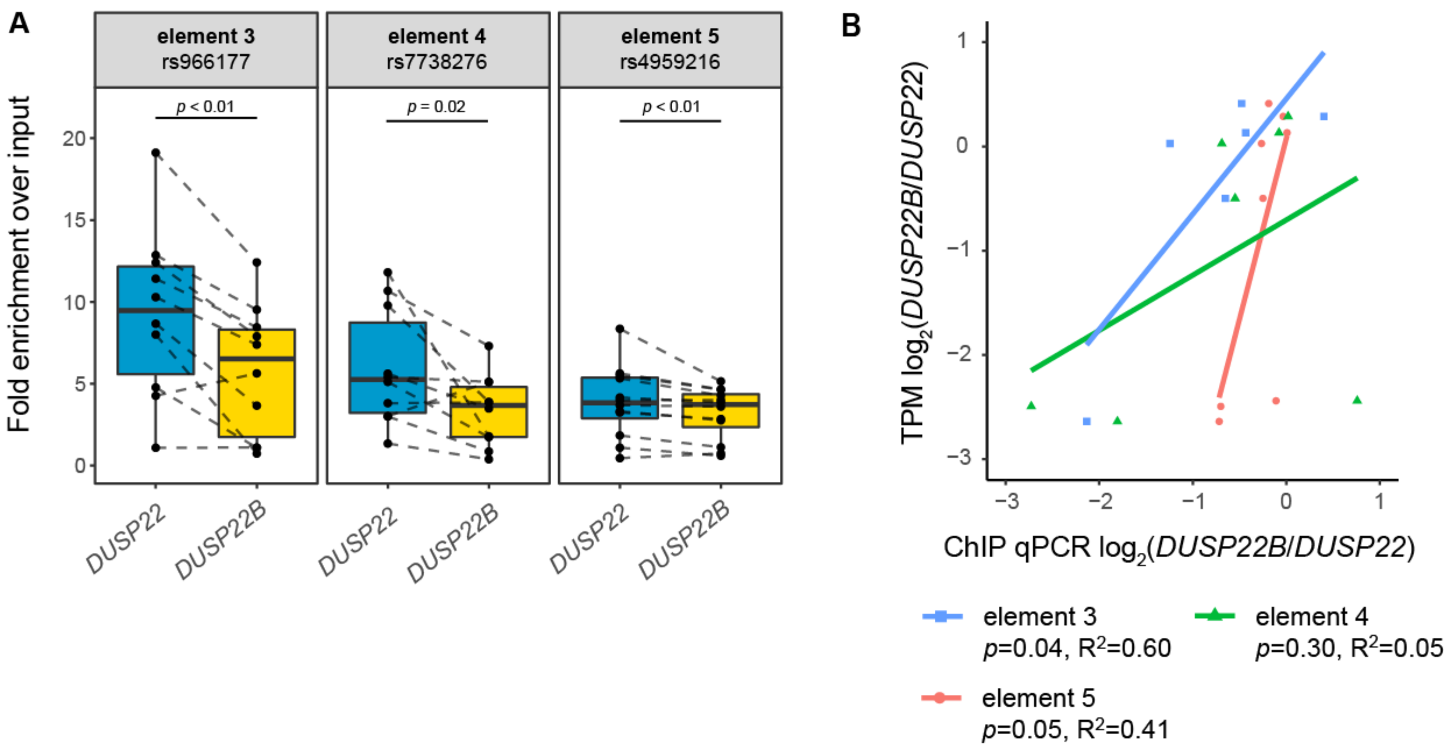

Figure S7. Paralogous differences of *DUSP22* H3K27ac peaks. To verify our H3K7ac ChIP-seq results in *DUSP22* and *DUSP22B*, as well as and assess biological reproducibility, we leveraged PSVs (annotated as SNPs in dbSNP) to perform paralog-specific ChIP-qPCR of three H3K27ac-enriched peaks in these genes. **(A)** *DUSP22* and *DUSP22B*-specific enrichment at three putative CREs, with paralogs distinguished using PSVs (N=12, 10, and 10 LCLs, respectively). Each variant lies within an element tested for enhancer activity with a luciferase reporter (Figures 5, S16, S17). Measurements were performed in triplicate and averaged. Differences in enrichment between paralogs were determined with a Wilcoxon signed-rank test, and *p*-values are denoted between the boxplots. **(B)** Correlation of expression divergence ($\log_2$ ratio of TPMs) with differences in enrichment (ChIP-qPCR signal) at the same three variants, for LCLs with RNA-seq data (Pickrell et al. 2010) (N=6, 7, and 8, respectively).

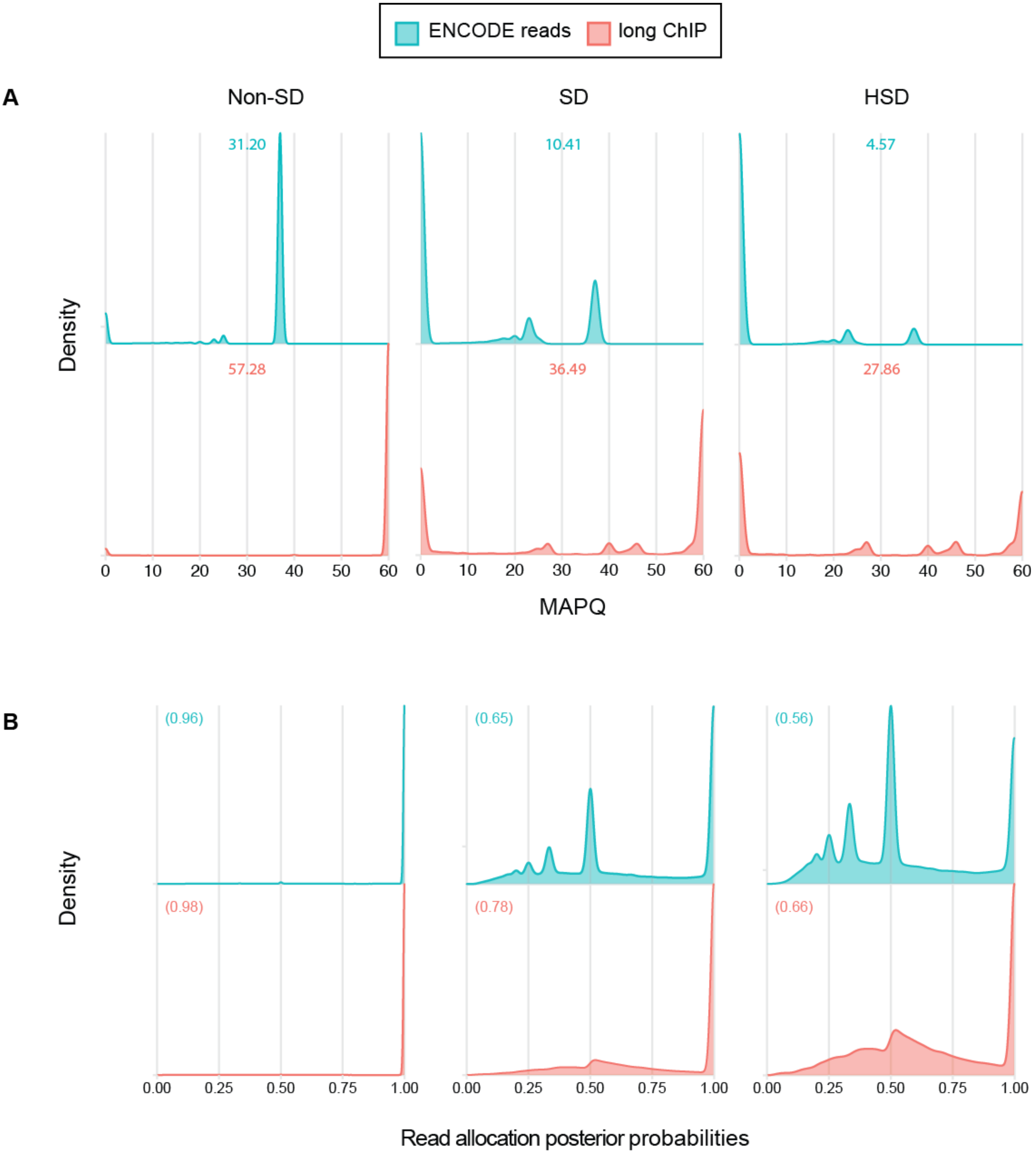

Figure S8. Summary of long ChIP data analysis (H3K27ac). Density plots are shown for the whole genome (left), SDs (middle), and SDs of over 98% sequence identity (HSD, left). **(A)** Distribution of BWA aln (ENCODE short reads, top) and BWA-MEM (long ChIP, bottom) alignment mapping quality (MAPQ) scores. The mean MAPQ score is shown on the top of each panel. **(B)** Distribution of bowtie (ENCODE short reads, top) and bowtie2 (long ChIP, bottom) CSEM posterior alignment probabilities. The mean posterior probability is shown on the top of each panel.

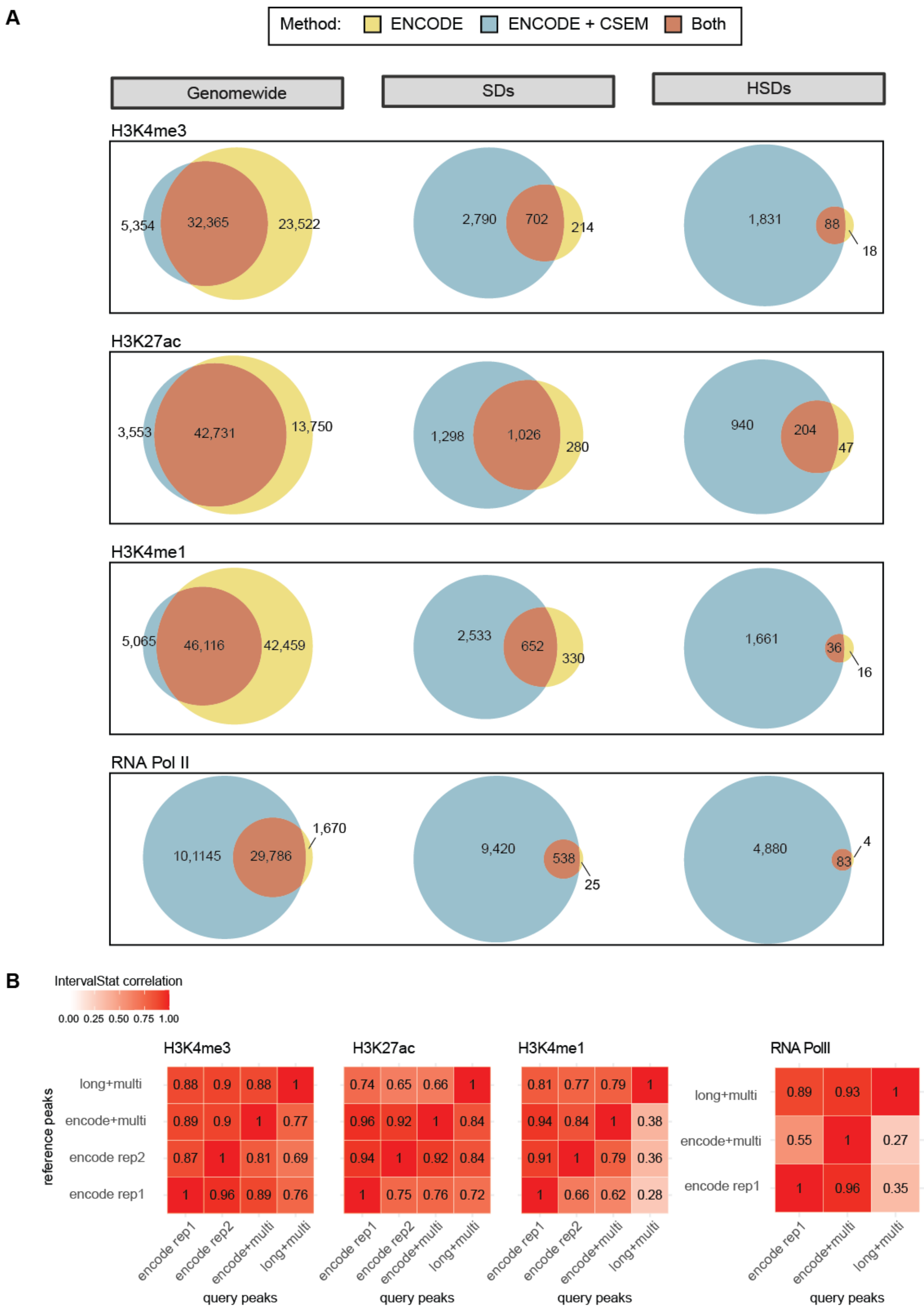

Figure S9. Comparison of ChIP-seq peaks from long ChIP data with multimapping and CSEM allocation and published ENCODE. (A) Overlap between data sets is shown for the whole genome, SDs, and HSDs (SDs of over 98% identity) for H3K4me3, H3K27ac, H4K4me1, and RNA PolII. Color indicates the method used for read mapping. **(B)** Pairwise correlations of genome-wide peak sets from single ENCODE replicates, ENCODE multi-mapping with CSEM allocation, and long ChIP multimapping with CSEM allocation. Unidirectional correlations were determined using IntervalStats (Chikina and Troyanskaya 2012), with overlapping peaks defined at $p<0.05$.

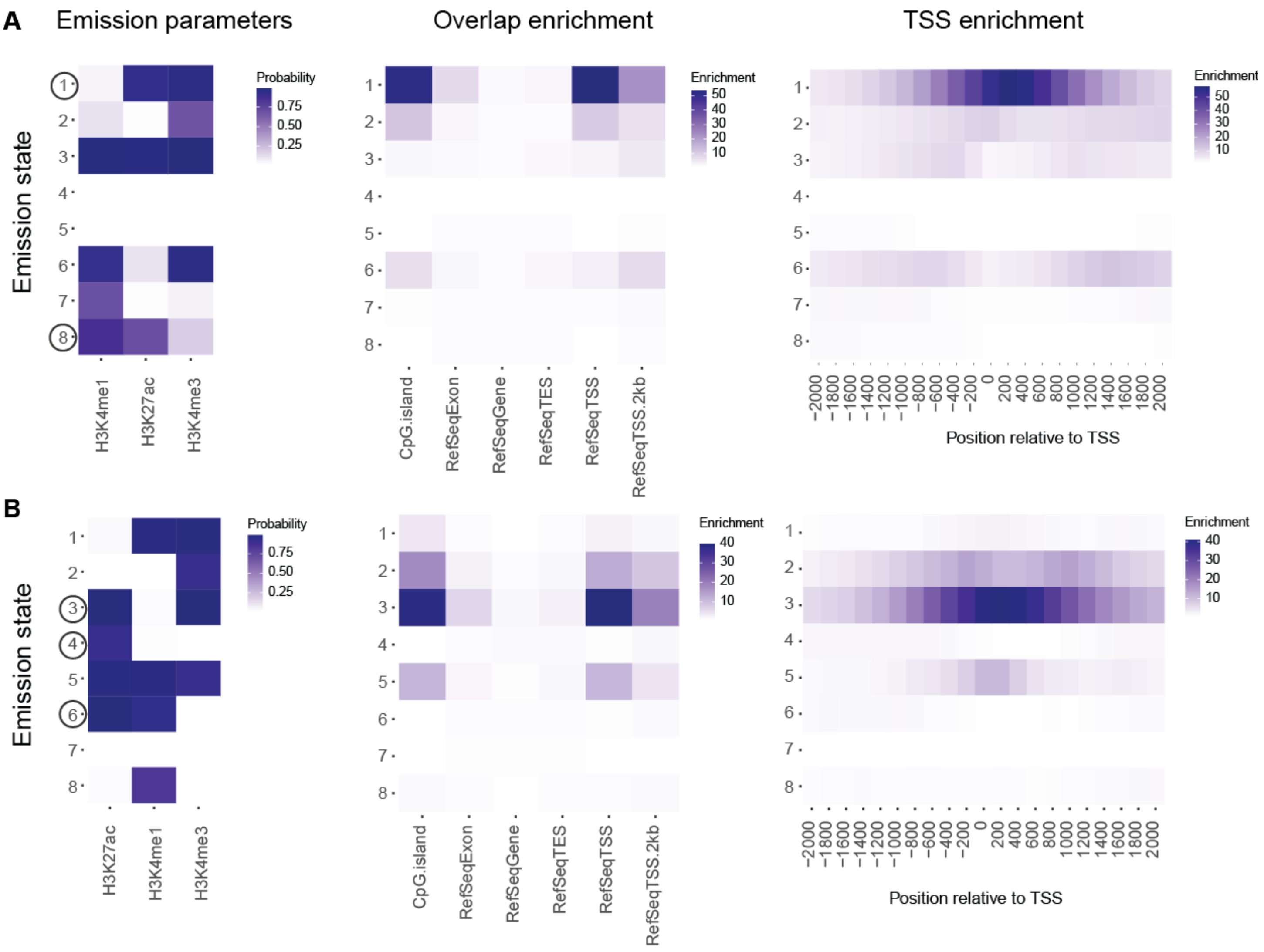

Figure S10. ChromHMM models used to cCREs. (A) Emission parameters, genomic features overlap, and enrichment relative to transcription start sites (TSSs) of an 8-state ChromHMM model built on ENCODE data (multiple-mapping and CSEM allocation). Darker blue indicates a higher probability of observing a given histone mark in each state, or a higher fold-enrichment in a given genomic feature or distance from TSS. State 1 was chosen to represent active promoters, and state 8 was chosen for active enhancers (circled). **(B)** Emission parameters, genomic features overlap, and enrichment relative to TSSs for an 8-state ChromHMM model built on long ChIP data (multiple-mapping and CSEM allocation). State 3 was chosen for active promoters, and states 4 and 6 were chosen for active enhancers (circled).

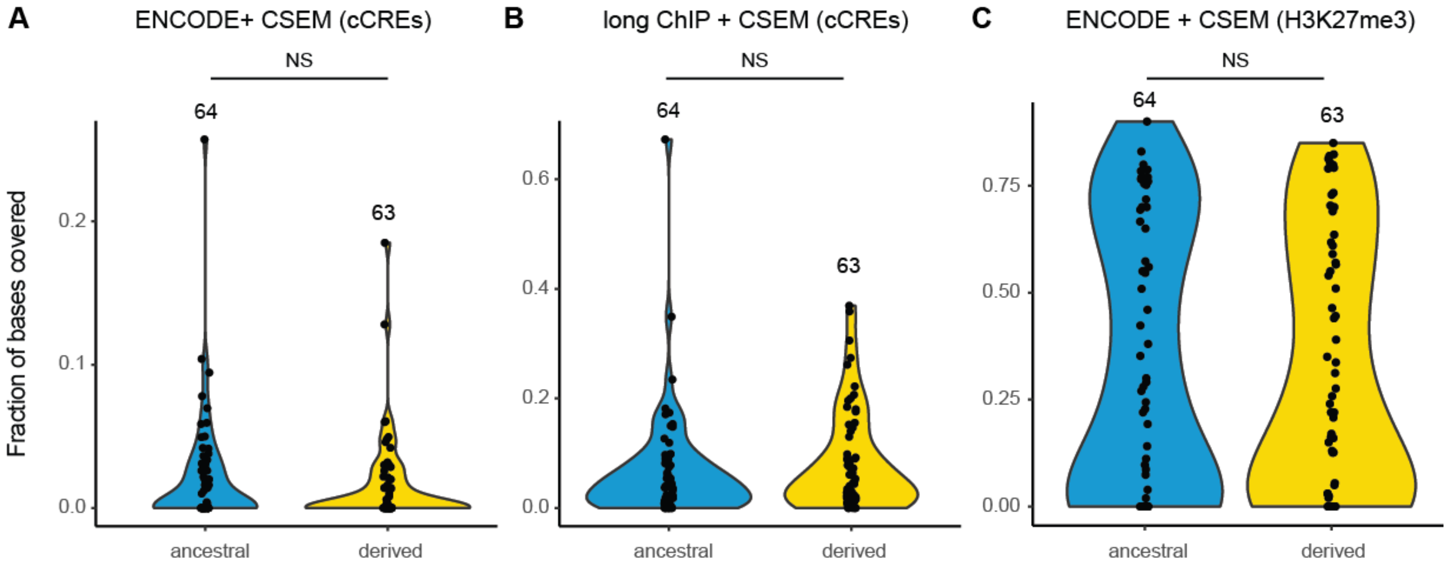

Figure S11. Global comparison of ancestral and derived HSDs. Violin plots represent the fraction of bases covered by (A) ENCODE multi-mapping cCREs, (B) long ChIP multi-mapping cCREs, and (C) ENCODE multi-mapping H3K27me3 domains. Fractional coverage was calculated in 100-kb windows for ancestral and derived HSD regions. Values were compared with a Wilcoxon signed-rank test.

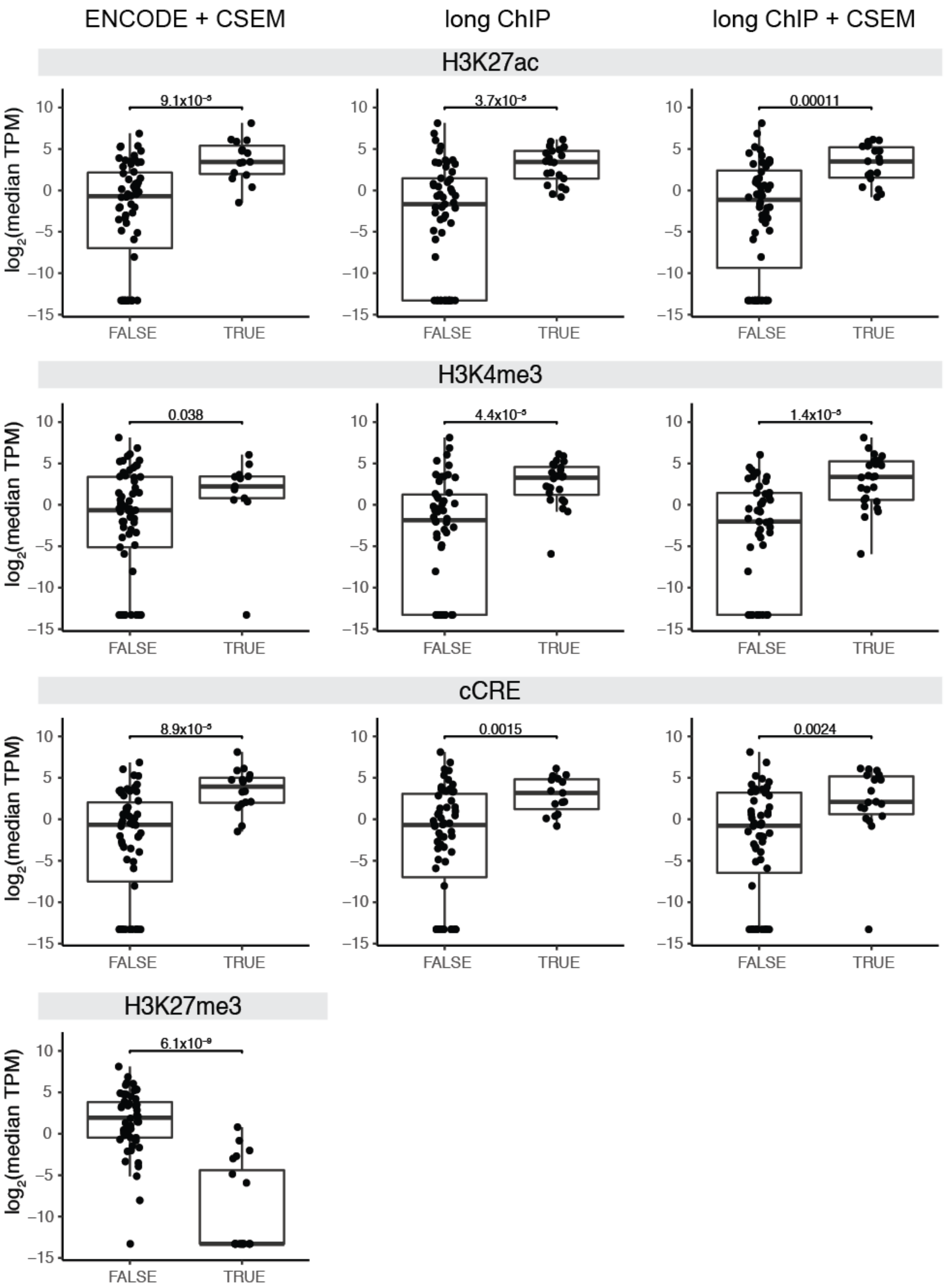

Figure S12. Associations of ChIP-seq marks and cCREs with HSD gene expression in LCLs. HSD genes were categorized by intersection of the transcription start site ("TRUE" or "FALSE") with a ChIP-seq peak or cCRE from the reanalyzed ENCODE data with multimapping and CSEM allocation (left), long ChIP data with single mapping (middle), and long ChIP data with multimapping and CSEM allocation (right). Expression values (TPM) are from LCLs from the Geuvadis consortium (Table S4; Lappalainen et al. 2013). *p*-values were generated from a Wilcoxon rank-sum test.

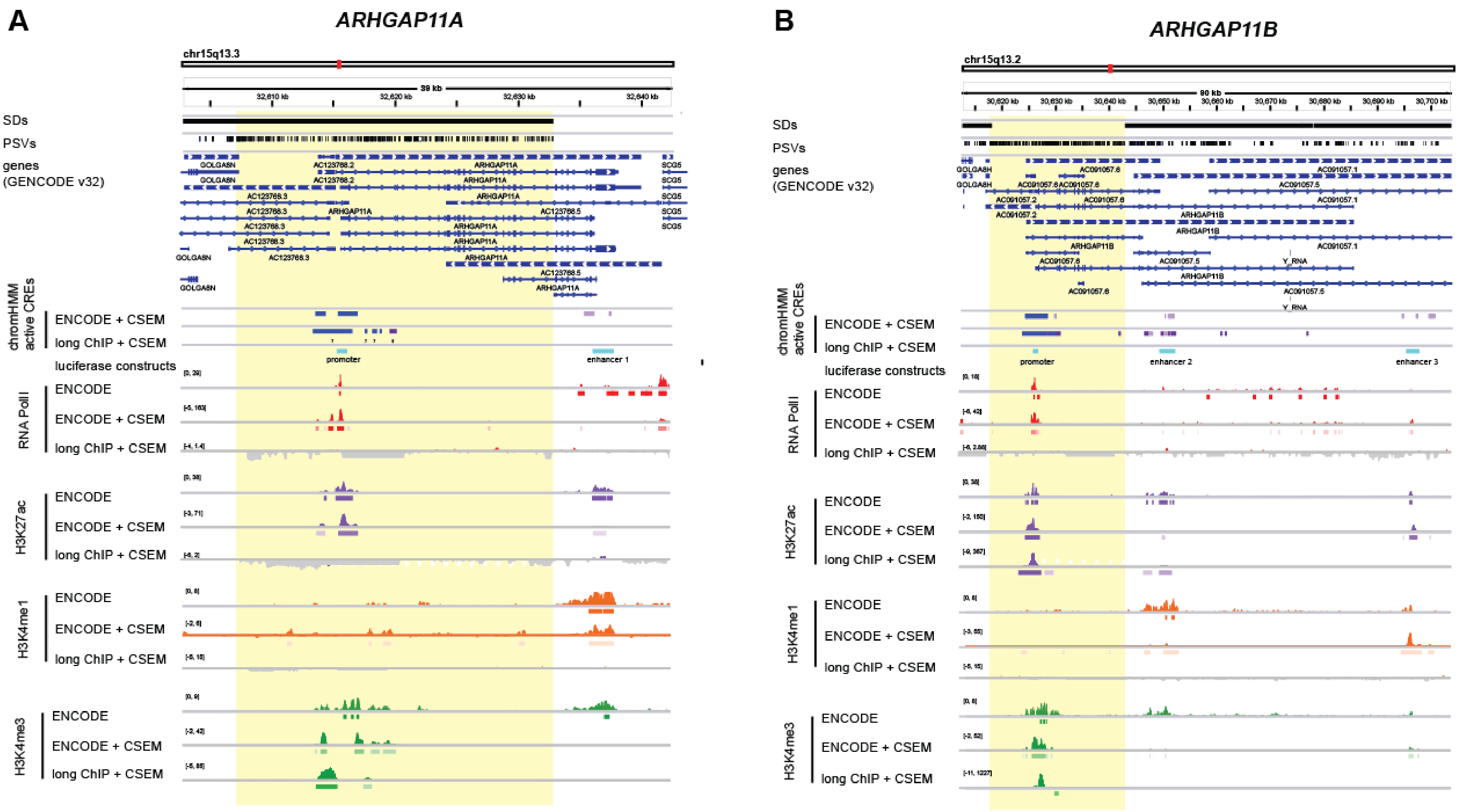

Figure S13. Epigenetic landscape of *ARHGAP11* genes. (A) *ARHGAP11A*. **(B)** *ARHGAP11B*. Coordinates indicate location on chromosome 15. The paralogous duplicated region is highlighted in yellow. Segmental duplications (SDs) and paralog-specific variants (PSVs) are indicated with black bars. ChromHMM segmentations are shown for active promoters (blue) and enhancers (lavender), as defined on ENCODE and long ChIP data (multimapping with CSEM allocation). Regions cloned and tested with luciferase reporters are shown in cyan. For each ChIP-seq target, a signal track is shown for published ENCODE; reanalyzed, multimapped ENCODE with CSEM allocation; and multimapped long ChIP with CSEM allocation. Visualized with the Integrative Genomics Viewer.

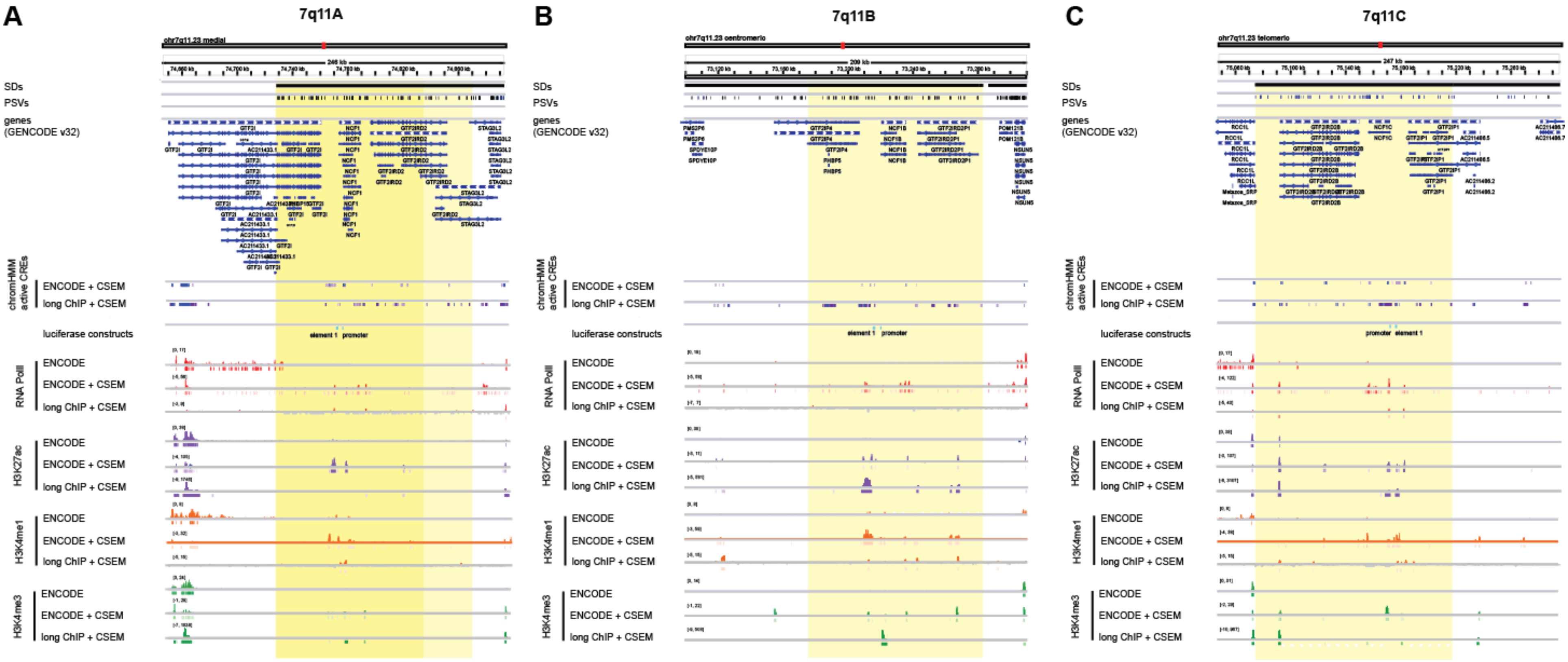

Figure S14. Epigenetic landscape of chromosome 7q11. (A) Ancestral locus. **(B)** Primary derived (centromeric) locus. **(C)** Secondary derived (telomeric) locus. Coordinates indicate location on chromosome 7. The paralogous duplicated regions are highlighted in yellow. Segmental duplications (SDs) and paralog-specific variants (PSVs) are indicated with black bars ChromHMM segmentations are shown for active promoters (blue) and enhancers (lavender), as defined on ENCODE and long ChIP data (multimapping with CSEM allocation). Regions cloned and tested with luciferase reporters are shown in cyan. For each ChIP-seq target, a signal track is shown for published ENCODE; reanalyzed, multimapped ENCODE with CSEM allocation; and multimapped long ChIP with CSEM allocation. Visualized with the Integrative Genomics Viewer.

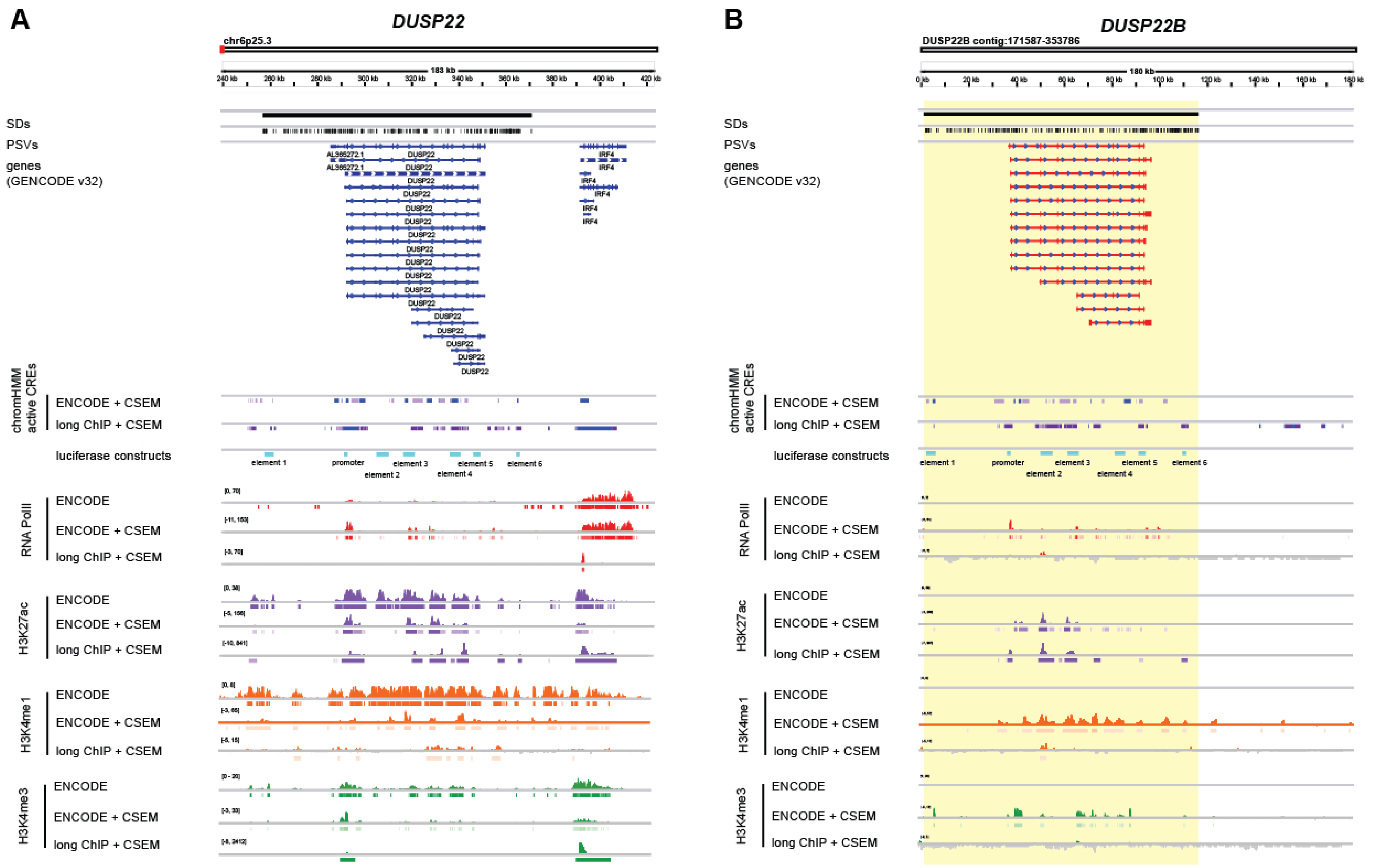

Figure S15. Epigenetic landscape of *DUSP22* genes. (A) *DUSP22*. **(B)** *DUSP22B*. Coordinates indicate location on chromosome 6 or the *DUSP22B* contig as published in (Dennis et al. 2017). *DUSP22B* gene models depict BLAT alignments of *DUSP22* transcripts. The paralogous duplicated region is highlighted in yellow. Segmental duplications (SDs) and paralog-specific variants (PSVs) are indicated with black bars. ChromHMM segmentations are shown for active promoters (blue) and enhancers (lavender), as defined on ENCODE and long ChIP data (multimapping with CSEM allocation). Regions cloned and tested with luciferase reporters are shown in cyan. For each ChIP-seq target, a signal track is shown for published ENCODE; reanalyzed, multimapped ENCODE with CSEM allocation; and multimapped long ChIP with CSEM allocation. Visualized with the Integrative Genomics Viewer.

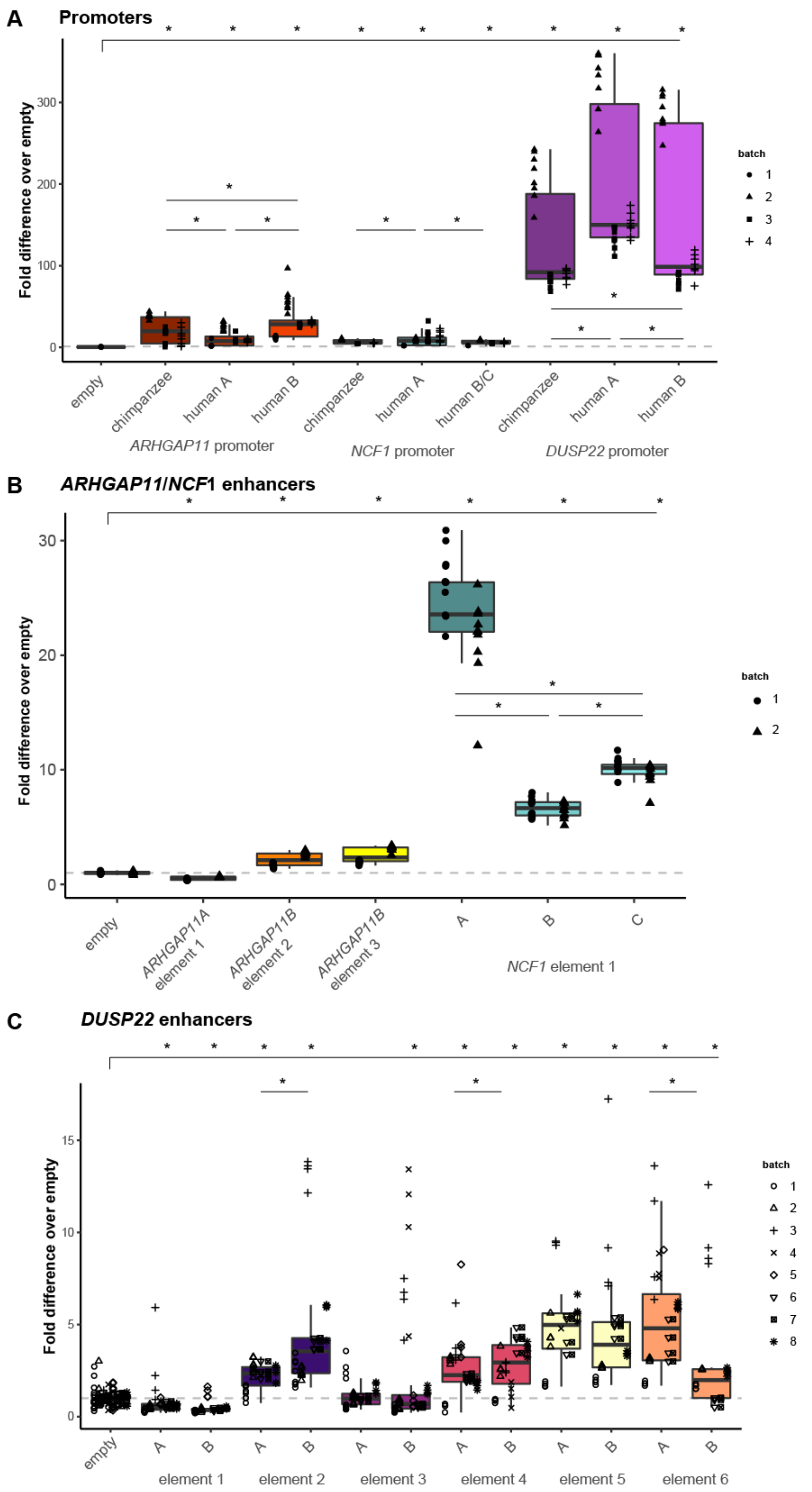

Figure S16 Luciferase activity of candidate CREs from all HeLa experiments. Luciferase activity for each cloned construct is shown as the fold difference over the average negative control value for **(A)** promoters, **(B)** *ARHGAP11* and *NCF1* candidate enhancers, and **(C)** *DUSP22* candidate enhancers. Values are visually separated by experimental batch. Significant differences ($p < 0.05$, Tukey post-hoc test of two-way ANOVA for batch correction) from empty (top bar) and between homologous sequences are indicated with an asterisk.

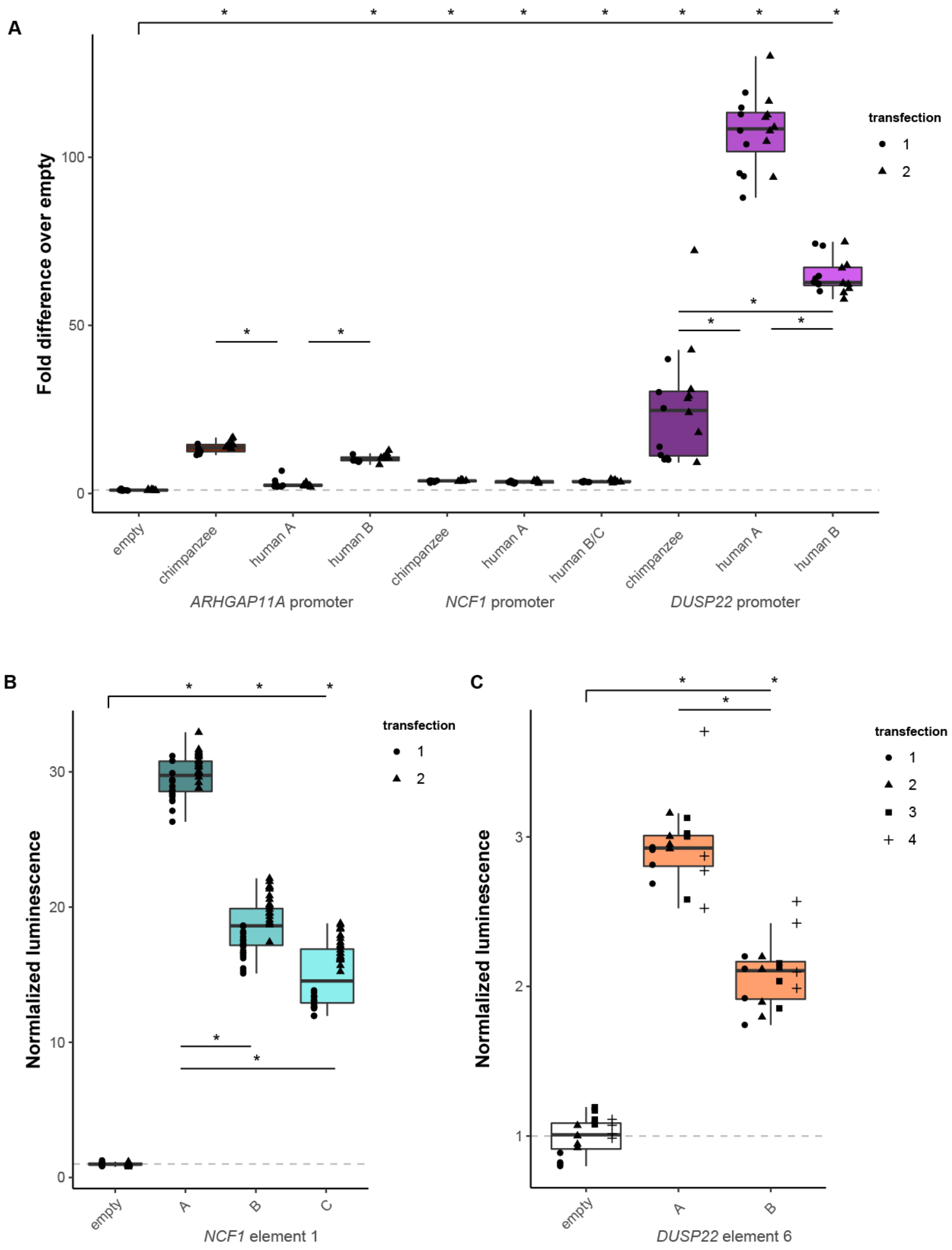

Figure S17. Luciferase activity of candidate CREs from all LCL experiments. Luciferase activity for each cloned construct is shown as the fold difference over the average negative control value for **(A)** promoters, **(B)** *NCF1* candidate element 1, and **(C)** *DUSP22* candidate element 6. Values are visually separated by experimental batch. Significant differences ($p < 0.05$, Tukey post-hoc test of two-way ANOVA) from empty (top bar) and between homologous sequences are indicated with an asterisk.

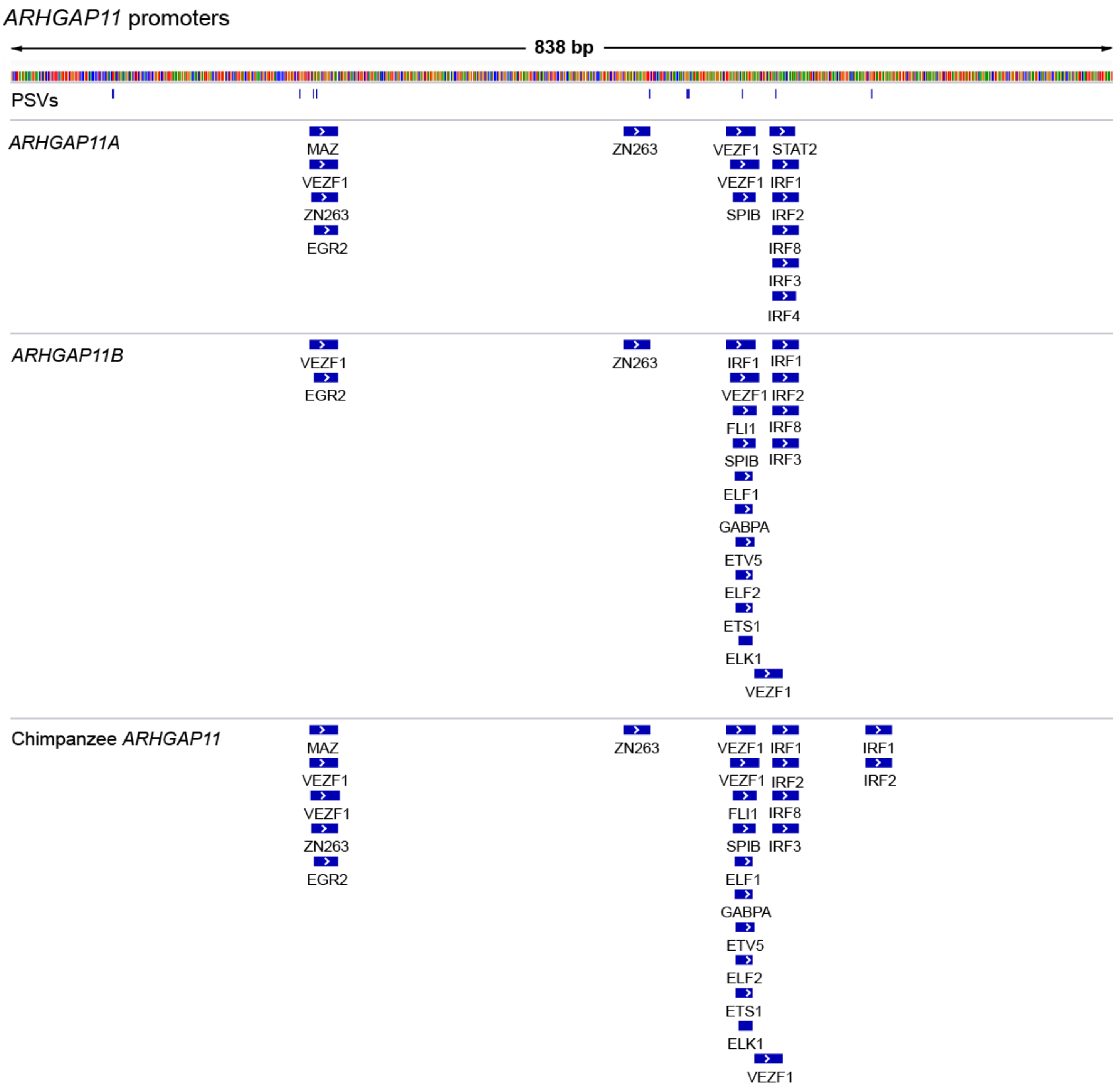

Figure S18. Transcription factor binding sites identified in *ARHGAP11* promoters. Significant matches (5% FDR) for HOCOMOCO v.12 Transcription factor binding site motifs intersecting PSVs are depicted under the cloned sequence tested with luciferase reporter. PSVs are depicted with blue vertical lines. Visualized with the Integrative Genomics Viewer.

Supplementary Note

We encountered a number of technical limitations while studying highly similar duplicated genes. We implemented an alignment-free approach for transcript quantification, which accommodates ambiguous mappings resulting from methods and is demonstrated to accurately distinguish highly similar transcripts (Soneson et al. 2015; Patro et al. 2017). However, reads originating from multiple transcripts are assigned probabilistically, and, as such, some genes may appear artificially similar in expression. For instance, *DUSP22B* expression was non-zero in individuals completely missing this paralog (Figure 3B) due to the recent nature of this duplication (<1 million years), which has resulted in very few PSVs differentiating the duplicates at the mRNA level. Conversely, RNA-seq quantification of older gene families (such *HIST2H2BF*) appeared entirely distinguishable between paralogs.

In addition, the available contigs of complete HSD loci have only been generated from a single haplotype (Dennis et al. 2017). As such, PSVs are not necessarily fixed, and may also be shared between genes as a result of interlocus gene conversion (Dumont 2015). Gene conversion can counteract the divergence of paralogous loci and has been predicted to prevent the fixation of neofunctionalizing variants (Teshima and Innan 2008). While we recognize the impact gene conversion may have on our results, a previous study by Dumont *et al* (2015) found only ~3% of SDs show signatures of interlocus gene conversion in the human genome, a result we recapitulated in our initial sequencing and evolutionary comparisons of these HSD genes (Dennis et al. 2017). There, we identified a European-specific gene conversion event between *CFC1* paralogs but were unable to assess its impact here as neither gene is expressed in LCLs. Increasing availability of multi-modal data (genomic and RNA) from diverse human tissues will allow us to better explore the impact of interlocus gene conversion on expression. Nevertheless, assessment of alleles within and between HSD paralogous loci is still prohibitively difficult with short-read sequencing.

As expected, we found that longer read lengths allowed greater bioinformatic distinction between duplicated loci, which remains an inherent limitation of many existing data sources, such as the relatively short reads (~30 nt) of ENCODE ChIP-seq. While longer reads are fundamentally more informative, some discrepancies between the ENCODE and long ChIP CSEM analyses could be a result of misallocation of reads. We were encouraged to find that a standard single-mapping approach (BWA MEM) identified novel peaks in HSD, albeit at a lower rate than genome-wide. As such, longer ChIP read lengths can be used in future studies to further describe the epigenetic state of duplicated loci with a broader array of epitopes and cell/tissue types.

Finally, though we used existing chromatin conformation information to connect non-duplicated adjacent regions with HSD gene promoters, improved experimental and bioinformatic methods are required to accurately link distal CREs within SDs to their target promoters. Like other genomic assays, available chromatin interactions from Hi-C data are sparse in SD regions due to poor mapping quality at similar paralogs. To our knowledge, one method exists (mHiC) that allocates multi-mapping reads to their possible alignments (Zheng et al. 2019), similar to CSEM. Future work might discover interactions within duplicated regions by reanalyzing chromatin conformation data in the most recent human reference (GRCh38) with this tool.

Supplementary Materials and Methods

Quantification of HSD gene expression

The following aligned Iso-Seq filtered alignments were obtained from the ENCODE portal (Davis et al. 2018) (https://www.encodeproject.org/): ENCFF225CCJ, ENCFF648NAR, ENCFF192PJS, ENCFF538BNH, ENCFF596ODX, ENCFF694CBG, ENCFF049QGQ, ENCFF846YHI, ENCFF600MGT, ENCFF810FRP, ENCFF479SQR, ENCFF504GVG, ENCFF731THW, ENCFF936VUF, ENCFF939EUU, ENCFF100RGC, ENCFF927MKK, ENCFF292UIE, ENCFF738RAA, ENCFF437SYY, ENCFF989FKA, ENCFF911RNV, ENCFF305AFY, ENCFF016SHE, ENCFF479EHE, ENCFF914XOH, ENCFF054YYA, ENCFF470UHX, ENCFF158KCA, ENCFF757LOZ, ENCFF809QBD, ENCFF779VVX, ENCFF971JDY, ENCFF319JFG, ENCFF901XCR, ENCFF117DUA, ENCFF772MSZ, ENCFF644PGG, ENCFF955XSL, ENCFF509GHY, ENCFF803KIA, ENCFF058HQU, ENCFF973OML, ENCFF745HHL, ENCFF472TSL. Reads were counted per HSD gene with HTSeq (Anders et al. 2015) before calculating RPKM values. For Figure S3B, *DUSP22* and *DUSP22B* reads were counted separately based on PSV sequence using SAMtools mpileup and the raw alignments in the following accessions: ENCFF132HLS, ENCFF234YIJ, ENCFF407TMX, ENCFF592BQN, ENCFF615XZM, ENCFF810UWA.

Human and chimpanzee RNA-seq data were quantified alignment-free with a custom reference transcriptome. Due to poor annotation of many HSD paralogs, custom transcriptomes were generated to ensure equivalent isoform models for paralogous genes, biasing against differential expression. First, transcript sequences for ancestral genes were extracted from GENCODE and mapped to derived human loci (contig from (Dennis et al. 2017) or GRCh38 for *SERF1B*) and a long-read chimpanzee assembly (Kronenberg et al. 2018) using BLAT (Kent 2002). GENCODE v27 transcripts were used for human-chimpanzee comparisons, since the chimpanzee transcriptome (Kronenberg et al. 2018) was built on this version; for human-only analyses, GENCODE v32 was used. Alignments were manually curated, and new derived transcripts were extracted from contigs. These transcripts, in addition to HSD transcripts generated from whole-isoform sequencing of brain tissue (Dougherty et al. 2018), were added to GENCODE (human) or the chimpanzee (after aligning to the chimpanzee assembly) transcriptome. Expression quantification was performed using Salmon v1.2.0 (Patro et al. 2017), the custom transcriptomes, and reference genomes (GRCh38 or Kronenberg et al. (2018)) as a decoy sequence. For paired-end data, we used the flags "--validateMappings" and "--gcBias". RNA-seq data were first lightly trimmed prior to quantification using trim_galore (https://github.com/FelixKrueger/TrimGalore) with the following flags: -q 20 --illumina --phred33 --length 20. Length-normalized TPM values or counts per gene were obtained using the tximport package in R (Soneson et al. 2015).

ChIP assays

ChIP assays were carried out as previously described with minor modifications (O'Geen et al. 2019). GM12878 cells were cross-linked in growth media containing 1% formaldehyde (Fisher Scientific BP531) for 10 min at room temperature and the reaction was stopped with 0.125 M glycine. Cross-linked cells were washed twice in PBS and stored at -80°C. 2×10^6 cells were used per ChIP assay. Cells from two biological replicates were lysed with ChIP lysis buffer (5 mM PIPES pH8, 85 mM KCl, 1% Igepal) with a protease inhibitor (PI) cocktail (Roche). Nuclei were collected by centrifugation at 2,000 rpm. for 5 min at 4°C and lysed in nuclei lysis buffer (50 mM Tris pH8, 10 mM EDTA, 1% SDS) supplemented with PI cocktail. Chromatin was fragmented in microTUBEs with the E220 (Covaris) using the low cell shearing protocol (Duty cycle 2%, PIP 105, CPB 200, 4 min) and diluted with 5 volumes of RIPA buffer (50 mM Tris pH 7.6, 150 mM NaCl, 1 mM EDTA pH8, 1% Igepal, 0.25% Deoxycholic acid). ChIP enrichment was performed by incubation for 16 h at 4°C with the following antibodies: 2 µg H3K27ac antibody (Active Motif #39133), 4 µg H3K4me1 antibody (Millipore 07-436), 2 µg H3K4me3 antibody (Active Motif #39915), or 2 µg RNA Polymerase II (PolII) antibody clone 8WG16 (Covance MMS-126R). RNA PolII samples were incubated for an additional hour with 2 µg Rabbit Anti-Mouse IgG (MP Biomedical #55436). Immune complexes were bound to 20 µl magnetic protein A/G beads (ThermoFisher) for 2 hours at 4°C. Beads were washed 2x with RIPA, 3x with ChIP wash buffer (100 mM Tris pH8, 500 mM LiCl, 1% Deoxycholic acid) and once with ChIP wash buffer plus 150 mM NaCl. ChIP samples were eluted in 100 µl ChIP elution buffer (50 mM $NaHCO_3$, 1% SDS) and cross-linking reversed with addition of 0.5 M NaCl and heating at 65°C overnight. Samples were treated with 2 µg RNaseA (Qiagen) and DNA was purified using the QIAquick PCR Purification Kit (Qiagen). ChIP enrichments were confirmed by qPCR with 2× SYBR FAST mastermix (KAPA Biosystems) using the CFX384 Real-Time System C1000 Touch Thermo Cycler (BioRad). ACTB primers served as positive control and HER2 primers as negative controls (Table S8). ChIP enrichment was calculated relative to input samples using the dC_t method (dC_t =

C_t[HER2-ChIP]-C_t[input]). Each entire ChIP sample was used to prepare Illumina sequencing libraries using the KAPA Hyper Prep Kit (Roche). Adapter-ligated DNA was separated on a 2% E-Gel EX (Invitrogen) and the 500–800 bp fraction was excised and purified using the QIAquick gel extraction Kit (Qiagen). Indexed primers were used to generate dual-indexed libraries and amplified libraries were size selected (500–700 bp) using the PippenHT (Sage Science). Equimolar library amounts were pooled and sequenced on the NovaSeq SP (Illumina).

Analysis of ChIP-seq data

ChIP-seq peaks obtained with the ENCODE pipeline were directly downloaded from the ENCODE portal (Davis et al. 2018) (https://www.encodeproject.org/) for H3K4me3 (ENCFF228GWY), H3K27ac (ENCFF367KIF), H3K4me1 (ENCFF453PEP), POLR2A (ENCFF455ZLJ), and H3K27me3 (ENCFF153VOQ). For "short" ChIP-seq peak calling using raw ENCODE data, GM12878 ChIP-seq reads were downloaded from the ENCODE portal for RNA Polymerase II (ENCSR000AKA), H3K4me3 (ENCSR000BGD), H3K4me1 (ENCSR000AKF), H3K27ac (ENCSR000AKC), and H3K27me3 (ENCFF000OBB). Illumina adapters and low quality bases (Phred score < 20) were trimmed using Trimmomatic (Bolger et al. 2014) (parameters SLIDINGWINDOW:4:20 MINLEN:20) and aligned to a custom reference genome (GRCh38 with an added *DUSP22B* contig) using single-end Bowtie (Langmead et al. 2009) configured to allow multiple mappings per read (parameters a -v2 -m99). After mapping, PCR duplicates were removed using Picard Markduplicates and secondary alignments were removed with SAMtools v1.9. Multi-mapping reads were allocated to their most likely position using CSEM v2.4 (Chung et al. 2011). CSEM was run using the --no-extending-reads option and the fragment size was calculated with phantompeakqualtools run_SPP.R script (Landt et al. 2012). A custom script was developed to select the alignment with the highest posterior probability as assigned by CSEM for each multi-mapping read, choosing one alignment randomly in case of a tie. Peaks were called using MACS2 callpeak (v2.2.6) on default settings using MACS2's shifting model (Zhang et al. 2008) (https://github.com/macs3-project/MACS). Broad peaks were called at a FDR of 5%, while narrow peaks were called at a FDR of 1%. BigWig files for peak's visualization were obtained with MACS2 bdgcmp tool and UCSC bedGraphToBigWig. For H3K27me3, which occurs in large domains, enriched regions were identified with hiddenDomains, using the default settings (Starmer and Magnuson 2016). Paired-end long-ChIP reads were generated as described above. Illumina adapters were removed using Trimmomatic (parameters SLIDINGWINDOW:4:30 MINLEN:50). Reads were mapped using both paired-end BWA-MEM and single-end Bowtie allowing for multiple mappings (parameters -a -n -S -e 200 -m 99). For single-end alignments, forward and reverse reads were concatenated into a single file and properly renamed to secure unique reads IDs. Reads aligned with BWA-MEM were filtered by MAPQ ≥ 20 while reads with multiple mappings aligned with Bowtie were allocated with CSEM and most likely alignments were selected with the custom script. Duplicates and secondary alignments were removed as explained above. Peaks were called using MACS2 with identical parameters used for short-reads, adding the BAMPE option in the case of paired-end reads aligned with BWA-MEM. Sets of peaks were compared between analysis methods using HOMER mergePeaks (parameters: "-d given") (Heinz et al. 2010) and a unidirectional correlation metric derived from IntervalStats using peaks with an overlap *p*-value below 0.05 (Chikina and Troyanskaya 2012).

Luciferase reporter assays

Promoters of highly and differentially expressed HSD gene families (*ARHGAP11*, *NCF1*, and *DUSP22*) were chosen for screening in a reporter assay. Fragments containing the TSS and spanning ~1 kb were amplified with KpnI and SacI restriction sites included in primers (Table S8) and cloned into the luciferase reporter vector pGL3-basic (Promega). Candidate enhancers within 50 kb of genes bodies were selected based on the presence of ChromHMM CREs in the re-analyzed data from human LCLs. Target regions were a maximum size of 5 kb, and peaks larger than this were tiled with multiple targets. Gateway homology arms were added to primers in accordance with the manual (ThermoFisher), and PCR products were cloned into the entry vector pDONR221 (ThermoFisher 12536017). Expression clones for luciferase assays were generated by cloning pDONR221 inserts into the luciferase reporter pE1B (Antonellis et al. 2008) with the Gateway system.

Constructs were co-transfected (ThermoFisher Lipofectamine 3000) in equimolar amounts with 50 ng of the control plasmid pRL-TK (Renilla luciferase) into HeLa cells in 96-well plates. Cells were at 70-90% confluence at the time of transfection. Luciferase assays were performed with the Dual-Luciferase Reporter Assay System (Promega E1910). 48 hours post-transfection, cells were washed with PBS, and lysed with Passive Lysis Buffer for at least 15 min sharking at 500 rpm. Lysates were stored at -80C. For LCLs, cells were split 48 and 24 hours pre-transfection to ensure active division. Cells were counted, washed in PBS, and resuspended such that each transfection contained 12.5×10^6 cells, 6.25

ug of test construct, and equimolar pRL-TK in RPMI. Cells were electroporated using the Neon Transfection System in accordance with previously published work (Tewhey et al. 2018) and recovered at a density of 3×10^6 cells/mL in pre-warmed RPMI including 15% FBS without antibiotics. Transfection efficiencies of ~15% were achieved. To perform luciferase assays, ~5×10^5 cells were pipetted into each well of a 96-well plate, washed with PBS, and lysed with Passive Lysis Buffer as described for HeLa. Luminescence measurements were performed according to the manufacturer's instructions using a Tecan Infinite or Tecan Spark plate reader with injectors.

References

Anders S, Pyl PT, Huber W. 2015. HTSeq--a Python framework to work with high-throughput sequencing data. *Bioinformatics* 31:166–169.

Antonellis A, Huynh JL, Lee-Lin S-Q, Vinton RM, Renaud G, Loftus SK, Elliot G, Wolfsberg TG, Green ED, McCallion AS, et al. 2008. Identification of neural crest and glial enhancers at the mouse Sox10 locus through transgenesis in zebrafish. *PLoS Genet.* 4:e1000174.

Blake LE, Roux J, Hernando-Herraez I, Banovich NE, Perez RG, Hsiao CJ, Eres I, Cuevas C, Marques-Bonet T, Gilad Y. 2020. A comparison of gene expression and DNA methylation patterns across tissues and species. *Genome Res.* 30:250–262.

Bolger AM, Lohse M, Usadel B. 2014. Trimmomatic: a flexible trimmer for Illumina sequence data. *Bioinformatics* 30:2114–2120.

Chikina MD, Troyanskaya OG. 2012. An effective statistical evaluation of ChIPseq dataset similarity. *Bioinformatics* 28:607–613.

Chung D, Kuan PF, Li B, Sanalkumar R, Liang K, Bresnick EH, Dewey C, Keleş S. 2011. Discovering transcription factor binding sites in highly repetitive regions of genomes with multi-read analysis of ChIP-Seq data. *PLoS Comput. Biol.* 7:e1002111.

Davis CA, Hitz BC, Sloan CA, Chan ET, Davidson JM, Gabdank I, Hilton JA, Jain K, Baymuradov UK, Narayanan AK, et al. 2018. The Encyclopedia of DNA elements (ENCODE): data portal update. *Nucleic Acids Research* 46:D794–D801. Available from: http://dx.doi.org/10.1093/nar/gkx1081

Dennis MY, Harshman L, Nelson BJ, Penn O, Cantsilieris S, Huddleston J, Antonacci F, Penewit K, Denman L, Raja A, et al. 2017. The evolution and population diversity of human-specific segmental duplications. *Nat Ecol Evol* 1:69.

Dougherty ML, Underwood JG, Nelson BJ, Tseng E, Munson KM, Penn O, Nowakowski TJ, Pollen AA, Eichler EE. 2018. Transcriptional fates of human-specific segmental duplications in brain. *Genome Research* 28:1566–1576. Available from: http://dx.doi.org/10.1101/gr.237610.118

Dumont BL. 2015. Interlocus gene conversion explains at least 2.7% of single nucleotide variants in human segmental duplications. *BMC Genomics* 16:456.

Ernst J, Kellis M. 2012. ChromHMM: automating chromatin-state discovery and characterization. *Nature Methods* 9:215–216. Available from: http://dx.doi.org/10.1038/nmeth.1906

Ernst J, Kellis M. 2017. Chromatin-state discovery and genome annotation with ChromHMM. *Nat. Protoc.* 12:2478–2492.

Fairley S, Lowy-Gallego E, Perry E, Flicek P. 2020. The International Genome Sample Resource (IGSR) collection of open human genomic variation resources. *Nucleic Acids Res.* 48:D941–D947.

Grant CE, Bailey TL, Noble WS. 2011. FIMO: scanning for occurrences of a given motif. *Bioinformatics* 27:1017–1018. Available from: http://dx.doi.org/10.1093/bioinformatics/btr064

Heinz S, Benner C, Spann N, Bertolino E, Lin YC, Laslo P, Cheng JX, Murre C, Singh H, Glass CK. 2010. Simple Combinations of Lineage-Determining Transcription Factors Prime cis-Regulatory Elements Required for Macrophage and B Cell Identities. *Molecular Cell* 38:576–589. Available from: http://dx.doi.org/10.1016/j.molcel.2010.05.004

Kent WJ. 2002. BLAT--the BLAST-like alignment tool. *Genome Res.* 12:656–664.

Khan Z, Ford MJ, Cusanovich DA, Mitrano A, Pritchard JK, Gilad Y. 2013. Primate transcript and protein expression levels evolve under compensatory selection pressures. *Science* 342:1100–1104.

Kronenberg ZN, Fiddes IT, Gordon D, Murali S, Cantsilieris S, Meyerson OS, Underwood JG, Nelson BJ, Chaisson MJP, Dougherty ML, et al. 2018. High-resolution comparative analysis of great ape genomes. *Science* 360. Available from: http://dx.doi.org/10.1126/science.aar6343

Kulakovskiy IV, Vorontsov IE, Yevshin IS, Sharipov RN, Fedorova AD, Rumynskiy EI, Medvedeva YA, Magana-Mora A, Bajic VB, Papatsenko DA, et al. 2018. HOCOMOCO: towards a complete collection of transcription factor binding models for human and mouse via large-scale ChIP-Seq analysis. *Nucleic Acids Res.* 46:D252–D259.

Landt SG, Marinov GK, Kundaje A, Kheradpour P, Pauli F, Batzoglou S, Bernstein BE, Bickel P, Brown JB, Cayting P, et al. 2012. ChIP-seq guidelines and practices of the ENCODE and modENCODE consortia. *Genome Res.* 22:1813–1831.

Langmead B, Trapnell C, Pop M, Salzberg SL. 2009. Ultrafast and memory-efficient alignment of short DNA sequences to the human genome. *Genome Biol.* 10:R25.

Lappalainen T, Sammeth M, Friedländer MR, 't Hoen PAC, Monlong J, Rivas MA, Gonzàlez-Porta M, Kurbatova N, Griebel T, Ferreira PG, et al. 2013. Transcriptome and genome sequencing uncovers functional variation in humans. *Nature* 501:506–511.

Law CW, Chen Y, Shi W, Smyth GK. 2014. voom: precision weights unlock linear model analysis tools for RNA-seq read counts. *Genome Biology* 15:R29. Available from: http://dx.doi.org/10.1186/gb-2014-15-2-r29

Marchetto MC, Hrvoj-Mihic B, Kerman BE, Yu DX, Vadodaria KC, Linker SB, Narvaiza I, Santos R, Denli AM, Mendes APD, et al. 2019. Species-specific maturation profiles of human, chimpanzee and bonobo neural cells. *eLife* 8. Available from: http://dx.doi.org/10.7554/elife.37527

Nguyen LS, Jolly L, Shoubridge C, Chan WK, Huang L, Laumonnier F, Raynaud M, Hackett A, Field M, Rodriguez J, et al. 2012. Transcriptome profiling of UPF3B/NMD-deficient lymphoblastoid cells from patients with various forms of intellectual disability. *Mol. Psychiatry* 17:1103–1115.

Noensie EN, Dietz HC. 2001. A strategy for disease gene identification through nonsense-mediated mRNA decay inhibition. *Nat. Biotechnol.* 19:434–439.

O'Geen H, Bates SL, Carter SS, Nisson KA, Halmai J, Fink KD, Rhie SK, Farnham PJ, Segal DJ. 2019. Ezh2-dCas9 and KRAB-dCas9 enable engineering of epigenetic memory in a context-dependent manner. *Epigenetics Chromatin* 12:26.

Patro R, Duggal G, Love MI, Irizarry RA, Kingsford C. 2017. Salmon provides fast and bias-aware quantification of transcript expression. *Nature Methods* 14:417–419. Available from: http://dx.doi.org/10.1038/nmeth.4197

Pavlovic BJ, Blake LE, Roux J, Chavarria C, Gilad Y. 2018. A Comparative Assessment of Human and Chimpanzee iPSC-derived Cardiomyocytes with Primary Heart Tissues. *Sci. Rep.* 8:15312.

Pickrell JK, Marioni JC, Pai AA, Degner JF, Engelhardt BE, Nkadori E, Veyrieras J-B, Stephens M, Gilad Y, Pritchard JK. 2010. Understanding mechanisms underlying human gene expression variation with RNA sequencing. *Nature* 464:768–772. Available from: http://dx.doi.org/10.1038/nature08872

Ritchie ME, Phipson B, Wu D, Hu Y, Law CW, Shi W, Smyth GK. 2015. limma powers differential expression analyses for RNA-sequencing and microarray studies. *Nucleic Acids Res.* 43:e47.

Shen F, Kidd JM. 2020. Rapid, Paralog-Sensitive CNV Analysis of 2457 Human Genomes Using QuicK-mer2. *Genes* 11. Available from: http://dx.doi.org/10.3390/genes11020141

Soneson C, Love MI, Robinson MD. 2015. Differential analyses for RNA-seq: transcript-level estimates improve gene-level inferences. *F1000Res.* 4:1521.

Starmer J, Magnuson T. 2016. Detecting broad domains and narrow peaks in ChIP-seq data with hiddenDomains. *BMC Bioinformatics* 17:144.

Teshima KM, Innan H. 2008. Neofunctionalization of duplicated genes under the pressure of gene conversion. *Genetics* 178:1385–1398.

Tewhey R, Kotliar D, Park DS, Liu B, Winnicki S, Reilly SK, Andersen KG, Mikkelsen TS, Lander ES, Schaffner SF, et al. 2018. Direct Identification of Hundreds of Expression-Modulating Variants using a Multiplexed Reporter Assay. *Cell* 172:1132–1134.

Zhang Y, Liu T, Meyer CA, Eeckhoute J, Johnson DS, Bernstein BE, Nusbaum C, Myers RM, Brown M, Li W, et al. 2008. Model-based analysis of ChIP-Seq (MACS). *Genome Biol.* 9:R137.
